# Low-Power Sputtered Cu_2_O Films with ∼70% Transmittance as a Possible Route toward Copper-Based p–n Junctions

**DOI:** 10.1021/acsmaterialsau.5c00255

**Published:** 2026-05-06

**Authors:** María del Pilar Aguilar-Del-Valle, Ana Laura Pérez-Martínez, Angélica Carrillo-Verduzco, Jesús Rafael González-Parra, Alejandra López-Suárez, Luis Fernando Garrido-García, José Reyes-Gasga, Arturo Rodríguez-Gómez

**Affiliations:** † Facultad de Ingeniería, DCB, 7180 Universidad Nacional Autónoma de México, Ciudad Universitaria, Coyoacán, Ciudad de México 04510, México; ‡ Instituto de Investigaciones en Materiales, Universidad Nacional Autónoma de México, Ciudad Universitaria, A.P. 70-360, Coyoacán, Ciudad de México 04510, México; § Centro de Ingeniería de Superficies y Acabados, Facultad de Ingeniería, Universidad Nacional Autónoma de México, Ciudad Universitaria, Coyoacán, Ciudad de México 04510, México; ∥ Instituto de Física, Universidad Nacional Autónoma de México, Circuito de la Investigación Científica s/n, Ciudad Universitaria,A.P. 20-364, Coyoacán, Ciudad de México 04510, México

**Keywords:** Copper Oxide, n-type Transparent Thin Films, Transparent Oxide Semiconductors, DC Sputtering, Oxygen vacancies, Optoelectronic devices

## Abstract

Developing stable copper-based transparent oxide semiconductors (TOSs) remains a scientific challenge, particularly via scalable processes that enable straightforward tuning of optical absorption, sheet resistance, and carrier type (p- vs n-type). Here we present a simple and reproducible methodology combining direct current (DC) sputtering and a thermal treatment to fabricate n-type copper-oxide TOS thin films. Ultrathin Cu layers deposited at 20 W in air and grown for 2–6 s on fused silica were converted by short-time annealing at 400 °C to single-phase Cu_2_O, enabling systematic modulation of thickness (39–105 nm) and average transmittance (69–45%, 400–700 nm). XPS confirmed a Cu- and O-dominated surface chemistry with no detectable nitrogen incorporation. The Cu 2p and Auger responses indicated a Cu­(I)-dominated chemical state, while the persistent high-binding-energy O 1s contribution after surface erosion was consistent with oxygen-related defective environments. Under our best-performing condition, the resulting Cu_2_O TOS exhibited an average transmittance of ∼ 70%, a resistivity of 34.2 Ω·cm, a sheet resistance of 8.76 × 10^6^ Ω/□, and a high-resolution Haacke figure of merit of Φ_*H*–*HR*_ = 0.1396, together with a bandgap of 2.2 eV and an electron concentration of 2.3 × 10^16^ cm^–3^. Building on our previous report of p-type copper-oxide transparent films (Cryst. Growth Des. 2022), these n-type Cu_2_O TOSs provide the complementary material platform toward copper-based p–n junction architectures using straightforward processing. Beyond junction-enabled concepts, the results offer a valuable proof of concept for stable n-type Cu_2_O, with potential applications in defect studies, sensors, and optoelectronic prototypes where moderate transparency and conductivity are acceptable.

## Graphical abstract

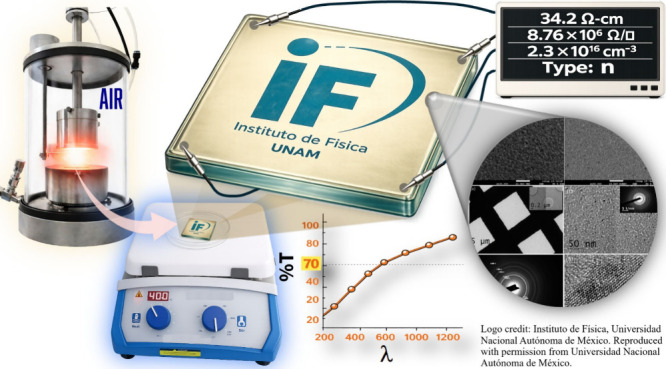

## 1 Introduction

Copper-based oxides are a versatile class of functional materials, with established relevance in catalysis, antimicrobial coatings, gas sensing, and optoelectronics.^1–8^ In particular, the prospect of copper-based transparent conductive and semiconducting films is attractive for emerging translucent electronics. However, achieving reliable control over optical transparency, electrical transport, and carrier type (p vs n) in copper oxides through scalable processing remains a central challenge.^9–17^

Optically transparent and electrically functional oxides are commonly realized as wide-bandgap metal oxides, often described within the broader family of transparent conductive oxides (TCOs) or transparent oxide semiconductors (TOSs).^18–20^ In many conventional TOSs, the valence band is dominated by localized O 2p states, which typically favors n-type transport and motivates extrinsic doping strategies to increase conductivity.^21–24^ Copper oxides, however, are a notable exception because hybridization between O 2p and Cu 3d states, often discussed as “chemical modulation of the valence band”, can facilitate p-type transport by enhancing hole density and mobility.^9,25–28^ Consequently, p-type copper-oxide TOS/TCO platforms are comparatively more accessible than their n-type counterparts.

Controlling carrier type in copper oxides is therefore technologically important. A stable and reproducible n-type copper-oxide layer would enable integration with the more accessible p-type copper-oxide transparent films, including those we previously reported,^9^ to advance copper-based p–n junction architectures for translucent devices. Yet, fabricating n-type copper oxide remains nontrivial, and reported strategies often depend on dopants or specialized deposition conditions.^29–32^ For example, Baturay and co-workers achieved n-type behavior in CuO via Zr doping,^33^ and in a related study, the same authors reported that Co incorporation influenced electron mobility.^34^ Similarly, Lakshmanan et al. demonstrated that cation doping can enable stable n-type Cu_2_O films deposited by industrially viable magnetron sputtering, using In or Al as dopants. Hall measurements showed a conductivity-type conversion from p- to n-type accompanied by a carrier density increase from ∼ 10^14^ to ∼ 10^17^ cm^–3^.^35^

In a different route, Yongli Du and collaborators obtained single-phase n-type CuO by DC magnetron sputtering under high-voltage, low-current deposition conditions.^36^ Along the same line of controlling growth kinetics, Farhad et al. reported single-phase Cu_2_O with either n- or p-type conductivity via pulsed laser deposition at low substrate temperatures (25–200 °C), where the carrier type and resistivity were tuned primarily through the background oxygen partial pressure and corroborated by Hall and Mott–Schottky analyses.^37^ More broadly, this result illustrates that, beyond extrinsic doping, identifying an appropriate deposition process window can also enable n-type transport in copper oxides.

The aforementioned studies underscore that the processing pathway strongly determines phase formation, defect chemistry, and transport in copper oxides. In this context, sputtering is attractive due to its control over composition and microstructure, its ability to produce uniform films with reduced contamination, and its compatibility with low thermal budgets.^38–40^ In addition, unlike solution-based approaches such as spray pyrolysis, spin coating, or electrochemical deposition, sputtering is a dry vacuum process that enables finer control over film thickness, interface quality, surface roughness, and lateral uniformity, all of which are essential for reproducible device fabrication. Compared with PLD, it also offers a more practical pathway toward large-area deposition and direct compatibility with established microelectronic and photovoltaic manufacturing technologies. Moreover, operating at low sputtering power can sustain stable discharges at low gas pressures, enabling thin films with controlled crystallinity and offering a practical handle to tune copper-oxide phase formation and, potentially, carrier type.^41,42^

Here, we investigate copper-oxide formation and optoelectronic transport using a deliberately simple strategy based exclusively on deposition parameters and a brief postdeposition thermal treatment, without extrinsic dopants or specialized processing. Starting from pure Cu films deposited by low-power DC sputtering, we find that deposition at 20 W followed by annealing at 400 °C promotes the formation of single-phase Cu_2_O with n-type behavior. Our best film exhibits a resistivity of 34.20 Ω·cm, an average transmittance of 69%, and an optical bandgap of 2.2 eV.

While the conductivity remains moderate, our results provide a reproducible proof of concept for stable n-type Cu_2_O and highlight that an appropriate sputtering and annealing process window can enable n-type transport in copper oxides. In combination with our previously reported p-type copper-oxide transparent films,^9^ the present findings define a materials-level route toward copper-based p–n junction architectures. At the same time, they underscore that realizing such junctions will require reconciling the distinct deposition and annealing windows needed to preserve the p-type and n-type responses during stacking. This platform is also relevant to defect-focused studies and to sensors or optoelectronic prototypes where moderate transparency and conductivity are acceptable.

## 2 Results

The results presented in the following subsections correspond to postannealed samples. Metallic Cu thin films were first deposited by DC sputtering from a pure Cu target under identical power conditions while varying the deposition time. All as-deposited Cu layers were then subjected to the same short-time thermal treatment at 400 °C (temperature profile shown in Figure 3(a)). Accordingly, the data discussed below refer to the Cu-oxide TOS films obtained after this standardized annealing step. All experimental details, including the deposition and annealing protocols, are provided in Section 5 (Experimental Section).

### 2.1 Atomic Force Microscopy Analysis and Thickness Measurements

Figure 1 shows the evolution of the surface topography of copper oxide films deposited for 2, 4, and 6 s, based on atomic force microscopy (AFM) measurements. Panel (a) presents the two-dimensional micrographs, all with an internal scale bar of 500 nm, showing that the three samples form continuous and completely covered surfaces, but with clear morphological differences in topographic texture. The film deposited for 2 s exhibits a coarser relief, with larger apparent lateral prominences and a more marked height contrast, while the 4- and 6-s samples show a finer, more compact, and homogeneous nanogranular texture.

**Figure 1:**
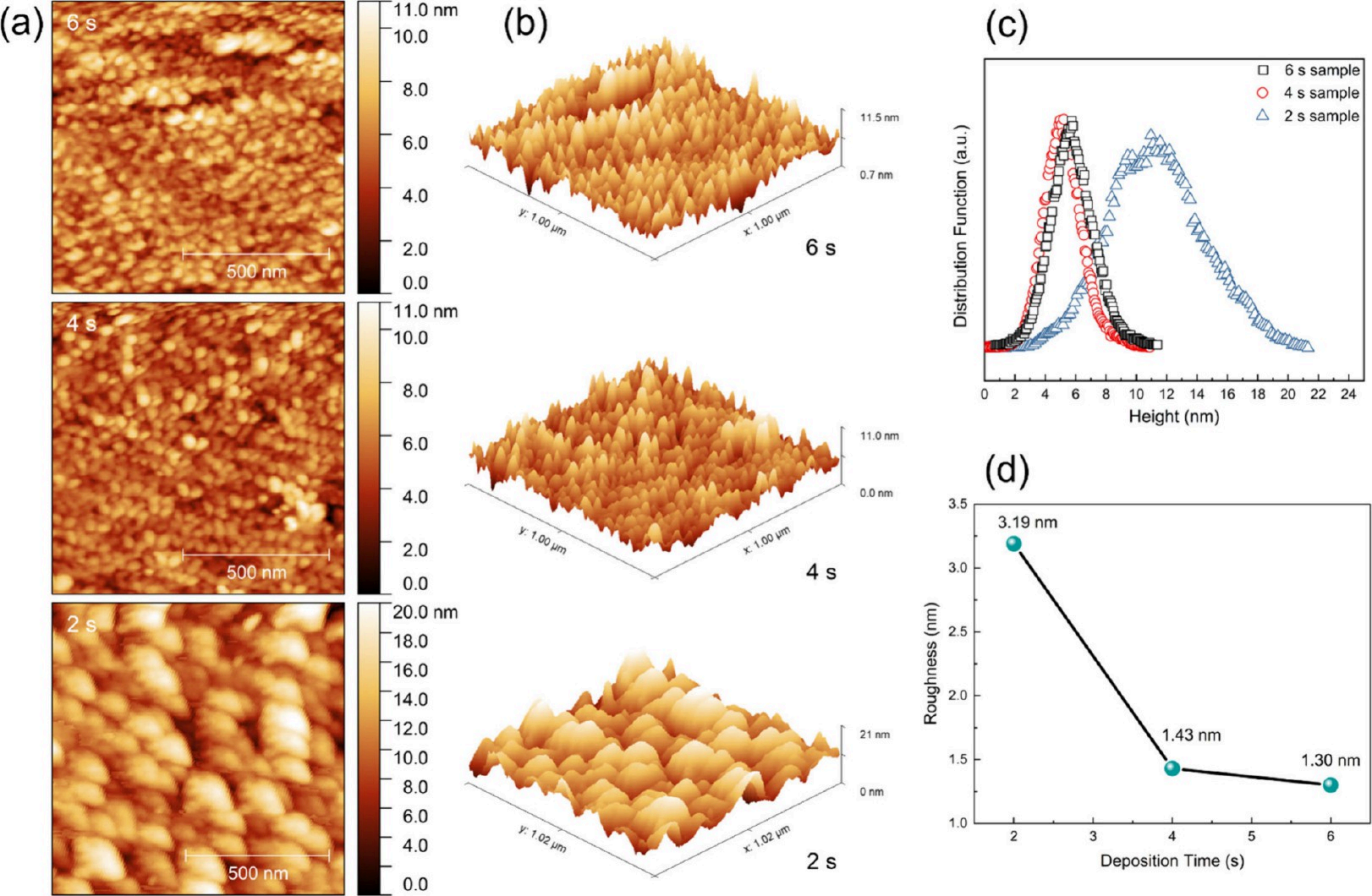
AFM characterization of copper oxide thin films deposited for 2, 4, and 6 s. (a) Two-dimensional topographic maps and (b) three-dimensional reconstructions, both showing a reduction in surface relief with increasing deposition time. (c) Height distribution functions for the 6 s (black squares), 4 s (red circles), and 2 s (blue triangles) samples, with the 2 s sample exhibiting a wider distribution shifted toward higher heights. (d) RMS roughness as a function of deposition time, with values of 3.19, 1.43, and 1.30 nm for 2, 4, and 6 s, respectively. The scale bars in (a) correspond to 500 nm.

This trend is confirmed in the three-dimensional reconstructions presented in Figure 1(b), where the 2-s sample shows a more pronounced vertical modulation and more developed surface mounds, in contrast to the 4- and 6-s films, whose topography becomes more uniform and smoothed. It should be noted that the vertical scale of the images is greater for the 2 s sample, reaching approximately 20–21 nm, while for the 4 and 6 s samples it remains around 11–11.5 nm, which already anticipates a substantial reduction in surface relief as the deposition time increases.

The AFM characterization results show that increasing deposition time leads to a marked attenuation of surface roughness, especially between 2 and 4 s, followed by a tendency toward stabilization between 4 and 6 s. This coincides with a transition from a more prominent, heterogeneous morphology to a nanometrically smoother, more homogeneous surface. A plausible explanation for the systematic decrease in roughness with increasing deposition time lies in the progressive attenuation of the substrate’s topographic contribution to the surface morphology measured by AFM. In most of the structural and morphological characterizations in this work, the films were deposited on conventional Corning glass obtained from commercial sample holders. The surface quality of this glass is inherently inferior to that of higher-purity, flatter optical substrates, such as the fused silica used exclusively for UV–vis spectroscopy measurements. Under these conditions, it is reasonable that in the thinnest film, corresponding to a 2 s deposition time, the recorded topography still retains a significant contribution from pre-existing irregularities, undulations, and defects in the substrate. As the thickness of the copper oxide layer increases, the film acquires greater capacity to cover, dampen, and mask this initial relief, so that the surface detected by AFM is increasingly governed by the film’s intrinsic morphology and, to a lesser extent, by that of the underlying substrate. Therefore, the reduction in roughness observed for 4 and 6 s is a natural consequence of more effective substrate coverage and a progressive topographic decoupling from the original substrate relief.

From a technological standpoint, the roughness values obtained, even for the 2 s film, remain in the low nanometric range, compatible with the integration of these layers into thin-film architectures. In particular, RMS roughness values of 3.19, 1.43, and 1.30 nm are sufficiently low for implementation in applications such as photovoltaic heterojunctions, thin-film diodes, thin-film transistors, and other multilayer optoelectronic configurations.^43–46^ Similarly, this level of topographic uniformity is favorable for the subsequent deposition of functional layers and for the formation of continuous interfaces, an essential requirement for establishing operational depletion regions in homo- or heterojunctions, provided that the composition, the electronic quality of the interface, and the band alignment are equally suitable. These AFM results support the viability of these films as active or interfacial layers within thin-film devices.

Accurate film thickness determination is indispensable for obtaining a reliable absorption coefficient (α), which is a key parameter for correctly estimating the optical bandgap. To achieve this, we employ a combined multitechnique approach. Initially, the step height defined by the Ni grid on the fused silica substrate is measured using a profilometer with a 10 nm resolution. Subsequently, thickness measurements are refined using topographic images obtained by Atomic Force Microscopy (AFM). In addition, cross-sectional Scanning Electron Microscopy (SEM) observations are incorporated as an independent and direct assessment of film thickness. The results obtained from these complementary methods are summarized in Table 1.

**Table 1:** Thickness Characterization of Thin Films As a Function of Deposition Time Using AFM, Profilometry, and SEM Cross-Section Techniques

Samples expressed in deposition time (s)	AFM thicknesses (nm)	Profilometer average thicknesses (nm)	Cross-sectional SEM thicknesses (nm)
2	40	39 ± 6	-
3	-	-	60.5 ± 0.8
4	65	68 ± 18	-
6	108	105 ± 9	140.9 ± 0.6

Figure 2 shows cross-sectional SEM micrographs used to verify the thickness of our TOS films. Since this work focuses on samples deposited for 2, 4, and 6 s, it is important to note that the 3 s sample was prepared exclusively for this characterization. This was because, for the 2-s sample, acquiring reliable cross-sectional images proved very difficult due to severe surface charging, which prevented clear definition of both the film/substrate interface and the film thickness. Therefore, the 3 s sample was chosen as the shortest deposition time for which the film could be adequately identified in cross-section.

**Figure 2:**
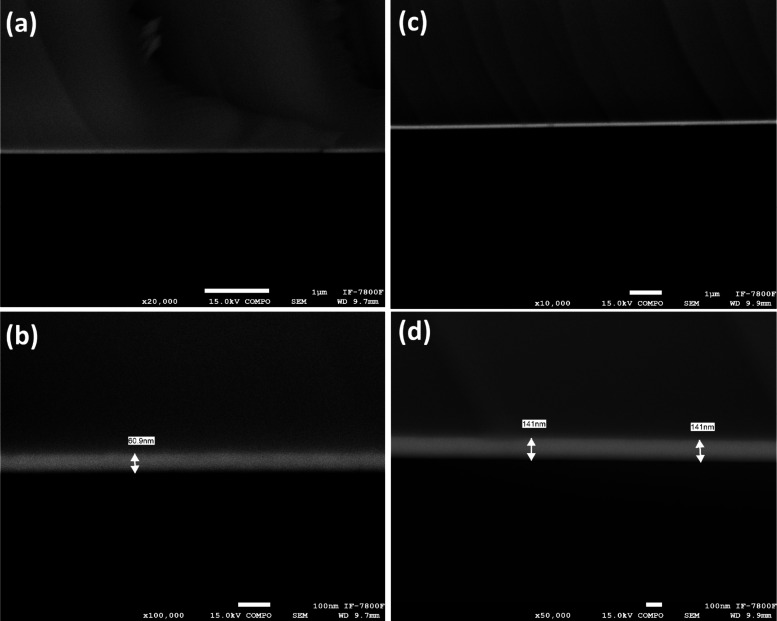
Cross-sectional backscattered-electron SEM micrographs of the Cu_2_O-based TOS films. Because the 2 s sample could not be reliably resolved in cross-section due to severe charging, a 3 s film was prepared specifically for this analysis. Panels (a,b) correspond to the 3 s film and show a continuous, laterally uniform layer with a thickness of 60.9 nm, whereas panels (c,d) correspond to the 6 s film and show a thicker layer with consistent local measurements of 141 nm.

Figure 2(a,b) corresponds to the 3 s sample. In (a), obtained at × 20,000 with a 1 μm scale bar, the general cross-sectional view of the sample is shown. In (b), at × 100,000 magnification and with a 100 nm scale bar, the film is clearly visible as a continuous, uniform band on the substrate, allowing a thickness measurement of 60.9 nm. Panels (c) and (d) correspond to the 6 s sample. In (c), acquired at × 10,000 magnification with a 1 μm scale bar, the film’s greater thickness allows it to be clearly distinguished even at lower magnification than in the 3 s sample. In (d), at × 50,000 magnification and with a 100 nm scale bar, the film is clearly identifiable, and consistent local measurements of 141 nm are obtained, demonstrating excellent lateral uniformity.

To evaluate the reproducibility of the cross-sectional SEM thickness measurements, two additional independent depositions were prepared and analyzed for the 3 and 6 s conditions. For the 3 s films, thicknesses of 60.9, 59.5, and 61.0 nm were measured, whereas for the 6 s films, thicknesses of 141.0, 140.3, and 141.5 nm were measured. These results yield average thicknesses of 60.5 ± 0.8 nm and 140.9 ± 0.6 nm for the 3 and 6 s samples, respectively, expressed as mean ± standard deviation for three independent depositions. These values correspond to deposition rates of 20.2 and 23.5 nm s^–1^ for the 3 and 6 s films, respectively, from which an overall average deposition rate of approximately 21.8 nm s^–1^ can be estimated.

To obtain a global yet conservative estimate of the growth kinetics, all available thickness measurements were converted into individual deposition rates by dividing the measured thickness by the corresponding deposition time, thereby integrating AFM, profilometry, and cross-sectional SEM data into a single framework. This treatment allows the incorporation of dispersion arising from both the measurement technique and variability among independent depositions into a single quantity, without imposing an artificial level of precision from any single method. From the complete set of individual rates, a global average deposition rate of (19.0 ± 2.3) nm · s^–1^ was obtained, where the uncertainty corresponds to the standard deviation of the values considered.

Within this approach, the thickness can be practically described as *d*(*t*) ≈ (19.0 ± 2.3)*t* with *d* in nm and *t* in seconds, explicitly showing that the uncertainty associated with thickness increases linearly with deposition time. This expression enables interpolation of expected thicknesses for intermediate growth times; for example, a thickness of 26.6 ± 3.2 nm is estimated for 1.4 s, whereas a value of 102.5 ± 12.5 nm is anticipated for 5.4 s. The proposed relation is not intended to replace a direct measurement, but rather to provide a reasonable quantitative reference for preliminary estimations.

### 2.2 Optical Characterization

The spectrophotometer’s limited spatial resolution allows the equipment beam to excite millions of copper oxide crystallites simultaneously. Therefore, we can comprehensively determine the thin film’s transmittance by measuring three different points on each sample. Additionally, we ensure the film’s optical uniformity by using this methodology and obtaining relatively similar spectra at the three different measurement points.

Figure 3(b) presents the transmittance spectra of our samples deposited for 2, 4, and 6 s, all subjected to the thermal treatment illustrated in Figure 3(a). Each spectrum shown is the average of three measurements taken from different areas of each sample. It is evident that in the region from 200 to approximately 380 nm, the transmittance remains below 20%. However, all three samples exhibit higher transmittance in the visible region of the electromagnetic spectrum, with values ranging between 40% and 60% at 532 nm and approaching 80% toward the end of the visible range. We must note that light absorption is a complex process involving several phenomena within the material. Therefore, in a typical absorption spectrum, the transmittance values are expected not to reach the extremes of zero or one hundred percent, just as seen in Figure 3(b).

**Figure 3:**
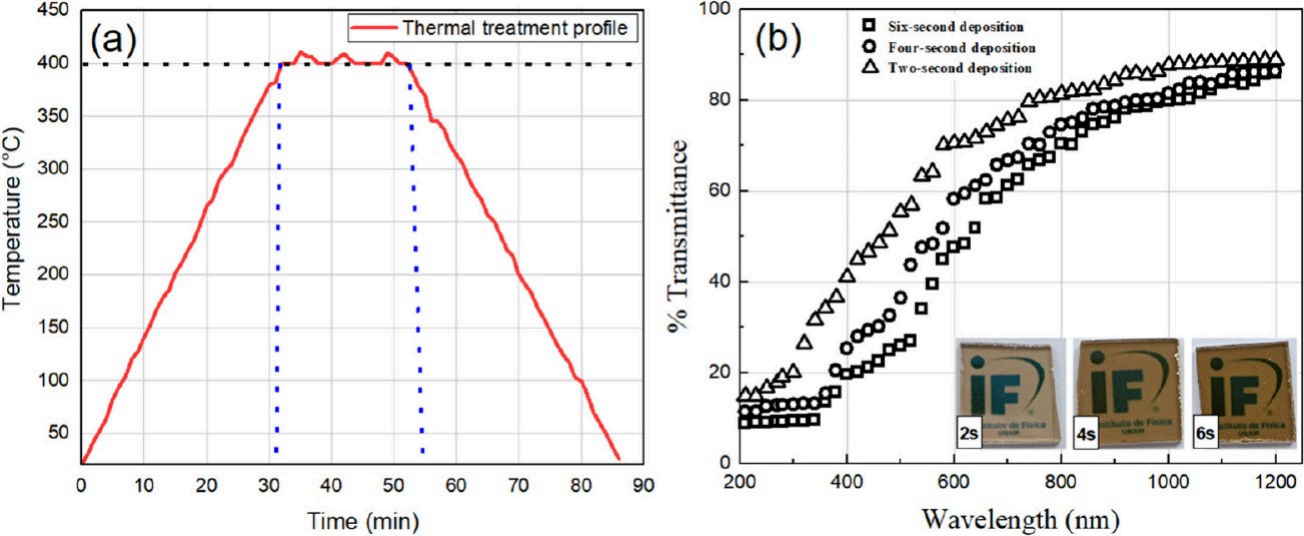
(a) Thermal treatment profile applied to the ultrapure copper films grown by sputtering. The short-time annealing step is performed from minute 32 to minute 52 at an approximately constant temperature of 400 °C. (b) Optical transmittance spectra of the copper-oxide-based TOSs obtained by depositing high-purity copper on fused silica and subsequently oxidizing the films using the thermal treatment shown in panel (a). The inset photograph shows the TOS produced from the 2, 4, and 6 s depositions after oxidation; the blue logo placed behind the sample is clearly visible through the coated substrate, providing a direct visual confirmation of high visible transparency. In this configuration, the logo remains distinguishable because a substantial fraction of the incident light traverses the film–substrate stack and, after reflection from the background, traverses it again, which is consistent with the relatively high transmittance measured across the visible and near-infrared ranges. Logo credit: Instituto de Física, Universidad Nacional Autónoma de México. Reproduced with permission from Universidad Nacional Autónoma de México.

Complementary to the film thickness measurements discussed in Section 2.1, the UV–vis-NIR spectrophotometric transmittance characterization presented in Figure 3(b) provides the second key experimental input required for determining the absorption coefficient (α). Under normal incidence, the amount of radiation absorbed by a material depends on its thickness, and each material has a characteristic absorption coefficient that varies with the wavelength of the incident light. Materials with higher α values are more prone to absorb electromagnetic radiation. This behavior is described by the Beer–Lambert-Bouguer law (T = *e* ^–αt^),^47^ where *T* represents the transmitted light fraction, α the absorption coefficient, and *t* is the sample thickness. Accurate knowledge of both transmittance and thickness enables the application of analytical models to estimate the optical bandgap with high confidence.

### 2.3 Determination of the Optical Bandgap

The transmittance spectra presented in Figure 3(b) can reveal valuable information about our copper oxide semiconductors, such as the states close to the valence and conduction bands and, of course, an estimation of the energy value that separates both bands. To obtain this information, specific data, particularly the absorption coefficient, must be extracted from the transmittance spectra, and then one of the semiclassical models should be applied to determine the optical bandgap.

Semiclassical models like Tauc’s,^48^ Davis-Mott’s,^49^ Cody’s,^50^ or Zanatta’s^51^ are so named because the charge carriers within the semiconductor receive a quantum mechanics treatment, while the electromagnetic radiation incident on the material is described solely in terms of the energy it delivers to the semiconductor. All the aforementioned semiclassical models use scatter plots that, in different ways, relate the absorption coefficient α to the energy of the incident photon *hv*, then, through curve fittings or extrapolations to the energy axis, the optical bandgap can be determined.

In this context, the most widely used semiclassical method to determine the optical bandgap (*E*_*B*_*g*__) is the Tauc model, which is expressed in eq 1,^40^ where *D* is a proportionality constant, *E*_*fot*_ = *hv* of the incident radiation, α is the semiconductor’s absorption coefficient, and *n* is 2 for allowed direct transitions and 1/2 for indirect transitions.^52^

1 $${\left(\alpha E_{fot}\right)}^{n}=D\left(E_{fot}-E_{Bg}\right)$$

Figure 4(a) shows the Tauc scatter plot of (α*hv*)^2^ against the incident photon energy for our three copper oxide semiconductors. For the 2-s deposition, an *E*_*B*_*g*__ of 2.18 eV is obtained, while for the 4 and 6-s depositions, values of 2.14 and 2.01 eV are, respectively, obtained. It is likely that the observed decrease in the optical bandgap in our copper oxide TOSs is not due to any change in the chemical composition or nanostructure of our deposition, as they were all deposited from the same copper target in the same batch, and all were subjected to the same annealing simultaneously. Because α was calculated from transmittance through the Beer–Lambert-Bouguer relation using the film thickness as an input parameter, the resulting Tauc plots are inherently sensitive to the thickness assigned to each sample. In this sense, the larger thickness values of the longer deposition-time films may contribute to lower calculated α values and, consequently, to slight variations in the optical bandgap extracted from the linear extrapolation.

**Figure 4:**
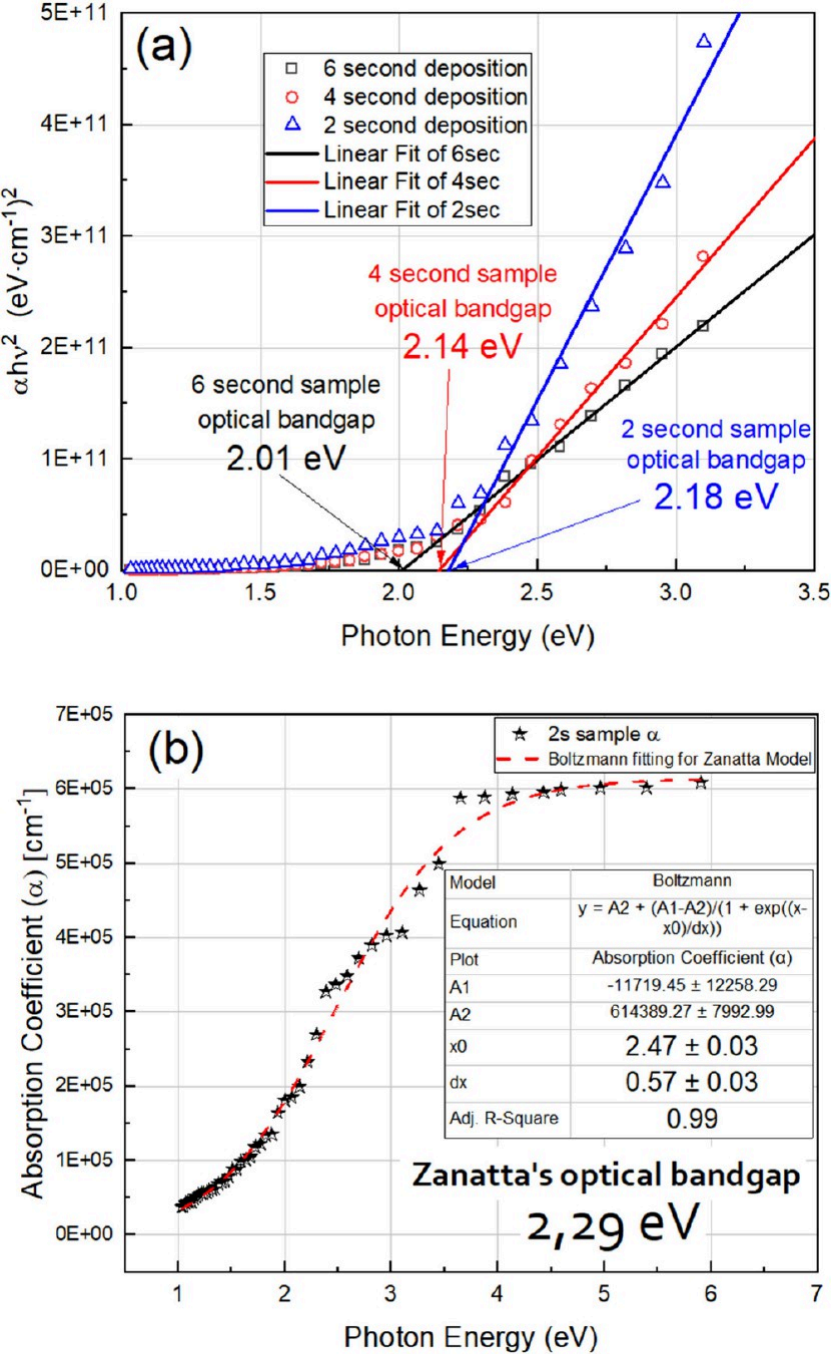
(a) Direct-transitions Tauc scatter plot for our three samples. The linear fitting for extrapolation was carried out at the interval from 2.6 to 3.1 eV. (b) Scatter plot of absorption coefficient against the photon’s energy for the 2-s sample subjected to short annealing. A sigmoid-Boltzmann fitting for the absorption coefficient is presented using a red dashed line.

It is important to note, however, that the uncertainty in the thickness of a given sample does not propagate significantly to the bandgap value determined by linear extrapolation on the Tauc plot. Since α is obtained as α = – (2.3026/t) · log_10_(T), a variation δt in thickness produces a proportional uncertainty δα/α = δt/t in the absolute values of α. Nevertheless, on the Tauc plot, the optical bandgap is determined as the ratio Eg = – A_1_/A_2_, where A_1_ and A_2_ are the intercept and slope of the linear fit of (αhν)^2^ versus hν, respectively. Since a uniform variation of α by a factor scales both A_1_ and A_2_ by the same factor, their ratio, and therefore Eg, remains invariant. This was verified numerically by recalculating the Tauc analysis for the 2 s sample with the extreme values t = 33 nm and t = 45 nm (corresponding to the ± 6 nm interval), obtaining in both cases an indistinguishable Eg value of 2.18 eV. The uncertainty in the thickness, therefore, mainly affects the absolute values of α and the magnitudes derived from these, but not the determination of the bandgap by linear extrapolation.

The apparent anomaly of having different bandgaps may be because all semiclassical methods for estimating the optical bandgaps are based on the optical absorption coefficient α­(E) derived from transmittance spectra. The subsequent analysis can be susceptible to artifacts such as sample thicknesses or even variations in climatic conditions during the transmittance measurement. Also, *E*_*B*_*g*__ values can differ by selecting a different range for the linear regression analysis. However, this last is not our case, as we have taken precisely the same ranges for extrapolation, as can be verified in Figure 4(a). In our case, the primary source of error that could generate the observed changes in *E*_*B*_*g*__ is the impossibility of accurately determining the thickness of the films. Our three TOSs thicknesses range between 40 and 140 nm with uncertainties that vary significantly between samples, as seen in Table 1.

However, the 2-s TOS stands out due to its lower absolute thickness and the significantly lower uncertainty associated with its measurement (±6 nm), which increases the reliability of any analysis dependent on this parameter. This contrasts with the 4-s deposition, which exhibits a much higher thickness uncertainty (±18 nm). In addition to this metrological advantage, the 2-s TOS also shows the highest average transmittance in the visible region, a highly desirable characteristic for its use as a translucent conductive contact.

A third physical consideration further reinforces the preeminence of the 2-s sample. Given its reduced thickness, it is reasonable to assume that oxygen could diffuse more efficiently during annealing, leading to a more homogeneous oxidation of the copper. This assumption is supported by a simple analogy: just as a bulk copper block typically oxidizes only at its surface while the interior remains metallic, thinner films are more likely to undergo complete oxidation throughout their volume. For all these reasons, we will henceforth consider the 2-s deposition as our flagship TOS for subsequent analyses.

Although the Tauc method has been widely used to estimate the optical bandgap, its scope is limited, as it does not provide access to other relevant material characteristics. In this work, we complement this estimation using the model proposed by Zanatta. Beyond refining the bandgap value derived from the Tauc approach, this method enables the evaluation of the degree of structural (dis)­order via parameters such as *δE*, offering a more comprehensive understanding of the material’s optical behavior.^51^

In the Zanatta model, a full fit of the absorption coefficient function α­(E) is performed using a Boltzmann-type sigmoid, as presented in eq 2

2 $$\alpha \left(E\right)=\alpha_{\max}+\frac{\alpha_{\min}-\alpha_{\max}}{1+e^{\left(E-E_{0}^{Boltz}/\delta E\right)}}$$

where α_*min*_ and α_max_ correspond to the minimum and maximum values of the absorption coefficient, *E* is the photon energy, *E*_0_^*Boltz*^ denotes the inflection point of the sigmoidal curve, interpreted as the characteristic energy above which most of the relevant optical transitions occur, and *δE* describes the energetic width of the transition, associated with the degree of structural (dis)­order in the material.

Figure 4(b) shows the absorption coefficient as a function of photon energy, α­(*E*), for our 2-s TOS, along with the corresponding fit using the Zanatta model. The sigmoidal curve provides an excellent fit to the experimental data, with an adjusted coefficient of determination of 0.99.

The inset in Figure 4(b) displays the fitting equation used by the analysis software: y = A2 + (A1-A2)/(1 + exp­((x-x0)/dx)). By comparing this expression with eq 2, a direct correspondence can be established between the parameters used by the fitting software and those in the original Zanatta model, namely α_*min*_ = A1, α_max_ = A2, *E*_0_^*Boltz*^ = x0, *δE* = dx. For our 2-s TOS, we identify *E*_0_^*Boltz*^ = 2.47 ± 0.03 eV and *δE* = 0.57 ± 0.03 eV.

From the parameters obtained through the sigmoidal fit in Figure 4(b), it is possible to apply the empirical formula proposed by Zanatta (eq 3) to estimate the optical bandgap of the semiconductor.

3 $$E_{Gap}^{Boltz}=E_{0}^{Boltz}-n_{dir-ind}^{Boltz}\times \delta E$$

This latter expression does not derive from a theoretical model but from a statistical analysis applied to more than 40 semiconductors, in which it was observed that *E*_0_^*Boltz*^ tends to overestimate the actual bandgap. Zanatta identified that this overestimation is correlated with *δE* and that the value of the coefficient *n* ^*Boltz*^ depends on the nature of the semiconductor; for direct-bandgap materials *n*_*dir*_^*Boltz*^ ≈ 0.3, while for indirect-bandgap materials *n*_*ind*_^*Boltz*^ ≈ 4.3 due to the need for phonon assistance to activate absorption.

Regarding the band structure of copper oxides, it has been reported that cuprous oxide (Cu_2_O) exhibits a direct bandgap, whereas cupric oxide (CuO) shows behavior characteristic of an indirect bandgap.^53^ This difference in the type of optical transitions is relevant for the interpretation of our results, since at this stage of the study, we have not yet presented experimental evidence that allows us to determine whether our thin films consist of a single copper oxide phase, either Cu_2_O, CuO, or a combination of both.

In this context, it is useful to apply the empirical formula from eq 3 using both *n*_*dir*_^*Boltz*^ and *n*_*ind*_^*Boltz*^ because, in addition to providing an estimated range for the bandgap, this approach allows a first inference as to whether direct or indirect band transitions govern the optical behavior in our film. Accordingly, by applying eq 3 to the parameters obtained for our 2-s TOS, we determine a direct *E*_*Gap*_^*Boltz*^ of 2.29 eV and an indirect *E*_*Gap*_^*Boltz*^ of 0.019 eV.

In a previous study, in which we reported the fabrication of cupric oxide films, applying the Zanatta model for an indirect bandgap yielded values of 1.92 and 1.87 eV.^9^ In those cases, structural and chemical characterization confirmed that most of the film consisted of cupric oxide. The marked difference from the present result (0.019 eV) suggests that our 2-s TOS is predominantly composed of a single cuprous oxide (Cu_2_O) phase, governed by direct band transitions, with a bandgap of 2.2 eV. This value is consistent with previous theoretical and experimental studies reporting Cu_2_O bandgaps in the range of 2.02 to 2.25 eV.^53,54^

Finally, within the framework of the Zanatta model, low *δE* values are associated with sharp absorption edges and highly ordered crystalline materials, whereas high values reflect increased disorder and the presence of extended states within the bandgap region. For instance, monocrystalline GaAs exhibits *δE* ≈ 0.01–0.02 eV, high-quality polycrystalline ZnO shows *δE* ≈ 0.15–0.30 eV, and amorphous oxides such as a-IGZO present *δE* ≈ 0.6–0.8 eV.^51^ Highly disordered materials, with mixtures of crystalline and amorphous phases or a high defect density, can reach values close to 1 eV. In this context, our value of *δE* = 0.57 ± 0.03 eV places the 2-s TOS at the upper limit of the range associated with polycrystalline materials, with a moderate degree of structural order and a relatively well-defined absorption edge, consistent with its observed optical transparency.

### 2.4 Hall Effect Measurements and Determination of the Figure of Merit

We quantified the performance of our TOS films using two key inputs, the average transmittance, *T*_avg_, and the sheet resistance, *R*_*s*_. The value of *T*_avg_ was obtained directly from the optical spectra discussed in Section 2.2 by averaging the transmittance over the 400–700 nm range. In turn, *R*_*s*_ was estimated via eq 4,^24^ using the resistivity ρ extracted from Hall-effect measurements and the film thickness *t* determined in Section 2.1. These quantities were then combined through Haacke’s high-resolution figure of merit, Φ_H–HR_, defined in eq 5,^56^ which provides a consistent basis to rank TOS quality and to compare samples on a quantitative scale. To ensure that the high-resolution Haacke figure of merit remains strictly consistent with the original Haacke formulation, an exponent of n = 10 must be used.

4 $$R_{s}=\frac{\rho }{t}$$

5 $$\Phi_{H-HR}=\frac{T_{avg}}{\sqrt[{n}]{R_{s}}}$$

Table 2 compiles, for our three samples, the Hall-effect electrical parameters measured at 100 μA (ρ, σ, carrier type, and *n*), together with *t*, *T*_avg_, the derived *R*_*s*_, and Φ_H–HR_. Among the conditions explored, the film deposited for 2 s exhibits the most favorable trade-off between sheet resistance and optical transmittance, resulting in the highest figure of merit among the TOSs produced in this work. Importantly, all samples preserve n-type conduction when employing a low sputtering power of 20 W.

**Table 2:** Summary of Thickness, Optical Transmittance, and Hall-Effect-Derived Electrical Parameters for Cu-Oxide TOS Thin Films Obtained from Cu Layers Deposited for 2, 4, and 6 s and Subsequently Converted by Short-Time Annealing at 400 °C^a^

Deposition time [s]	TOS Thickness [nm]	TOS Average Transmittance (%)	Hall effect applied current [μA]	Resistivity [Ω·cm]	Sheet Resistance [Ω/□]	Φ_*H*–*HR*_ [Ω^–1/10^]	Conductivity [S/cm]	Charge carrier concentration [*e*/*c* *m* ^3^]	Semiconductor Type
2	39	69	100	34.20	8.76 × 10^6^	0.1396	2.92 × 10^–2^	(2.3 ± 0.29) × 10^16^	N
4	68	56	100	53.69	6.8 × 10^6^	0.1161	1.86 × 10^–2^	(5.7 ± 1.03) × 10^16^	N
6	105	45	100	62.20	7.87 × 10^6^	0.0919	1.60 × 10^–2^	(1.2 ± 1.29) × 10^15^	N

a Tavg corresponds to the average transmittance in the 400–700 nm range. Hall measurements were performed in the Van der Pauw configuration using an applied current of 100 μA; the reported carrier concentration is given as mean ± standard error of the mean (*n* = 5).

One of the most debated issues regarding n-type copper-oxide TOSs is the reliable determination of the majority carrier type.^57–59^ To address this point, we prepared five independent films for each deposition time (2, 4, and 6 s), all processed under the same short-time annealing protocol. The replicate samples were not fully characterized in every respect; however, each one was carefully evaluated by Van der Pauw Hall-effect measurements. In all cases, n-type conduction was consistently observed, while the extracted carrier concentration showed some dispersion among replicates. Therefore, the values reported in the “charge carrier concentration” column correspond to the mean ± standard error of the mean.

### 2.5 Mott–Schottky Capacitance Analysis

Mott–Schottky (M-S) analysis is an electrochemical tool used to characterize semiconductor-electrolyte interfaces. It provides information on the nature of the majority carrier, the apparent density of ionized dopants, and the flat-band potential from the system’s capacitive response. Ideally, the 1/C^2^ plot of the applied potential exhibits linear behavior, with a positive slope for n-type semiconductors and a negative slope for p-type semiconductors. For an n-type semiconductor, this relationship is expressed by eq 6

6 $$\frac{1}{C^{2}}=\frac{2}{\varepsilon_{r}\varepsilon_{0}A^{2}eN_{d}}\left(V-V_{fb}-\frac{k_{B}T}{e}\right)$$

where *C* is the capacitance associated with the space charge region, *ε*_*r*_ is the relative permittivity of the semiconductor, *ε*_0_ is the permittivity of free space, *A* is the active area, *e* is the elementary charge, *N*_*d*_ is the apparent donor density, *V* is the applied potential, *V*_*fb*_ is the flat-band potential, *k*_*B*_ is the Boltzmann constant, and *T* is the absolute temperature. Experimentally, the methodology consists of plotting 1/*C* ^2^ as a function of *V* and analyzing the slope of the linear region; the slope’s sign identifies the type of conduction. Extrapolation to the potential axis allows estimation of *V*_*fb*_, correcting for the thermal term *k*_*B*_ *T*/*e*. Furthermore, the value of the slope can be used to estimate the donor or acceptor density, depending on the type of semiconductor analyzed.

To independently confirm the n-type conductive nature of our 2-s TOS, we performed a Mott–Schottky capacitance analysis in a 0.1 M Na_2_SO_4_ solution. The corresponding results are presented in Figure 5.

**Figure 5:**
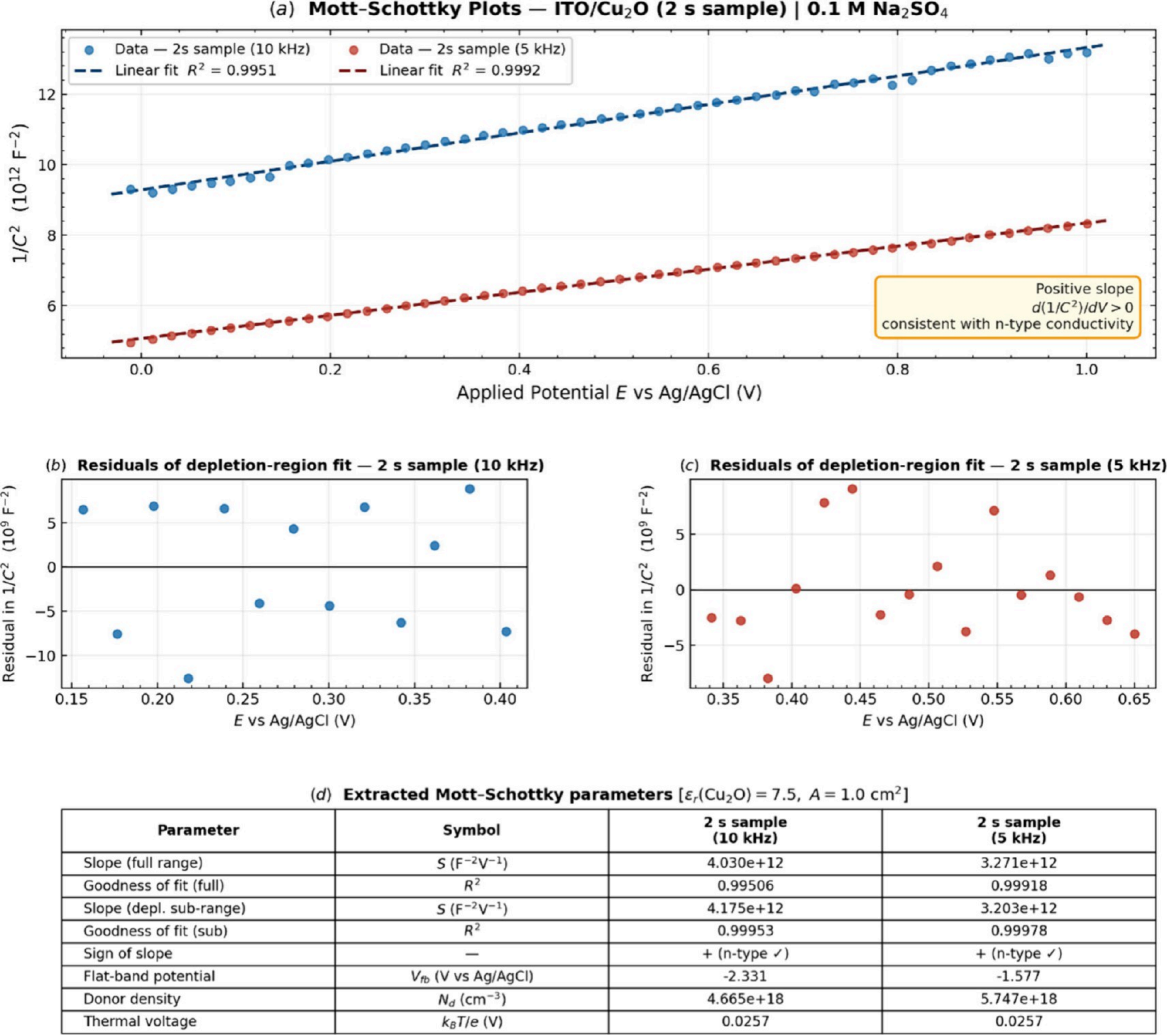
Mott–Schottky (M-S) analysis of the ITO/Cu_2_O film deposited for 2 s, measured in a 0.1 M Na_2_SO_4_ solution. (a) Plot of 1/C^2^ as a function of the applied potential E vs Ag/AgCl for two excitation frequencies, 10 and 5 kHz. In both cases, a linear relationship is observed over the explored potential range, with a positive slope. The dashed lines correspond to linear fits over the entire measurement range, with the correlation coefficients indicated in the legend. (b) and (c) Residuals of the linear fits performed in the depletion region for the measurements at 10 and 5 kHz, respectively. The distribution of the residuals around zero confirms the validity of the linear model within the selected parameter-extraction intervals. (d) Quantitative summary of the parameters obtained from the M-S analysis. The slopes and *R* ^2^ values corresponding to both the full interval fit and the fit in the depletion region are included, along with the sign of the slope, the flat band potentials *V*_*fb*_, the apparent donor densities *N*_*d*_, and the value of the thermal term *k*_*B*_ *T*/*e*. The values of *V*_*fb*_ and *N*_*d*_ were estimated from the linear extrapolation of the M-S curves, using *ε*_*r*_(Cu_2_O) = 7.5 and an active area of 1.0 cm^2^.

Figure 5(a) shows the curves of 1/C^2^ as a function of the applied potential E versus Ag/AgCl, recorded at 10 and 5 kHz. In both cases, linear behavior is observed across the explored potential range, with well-defined positive slopes. The sign of the slope d­(1/C^2^)/dV > 0 corresponds to that expected for semiconductors whose transport is dominated by electrons. In addition to the qualitative agreement between the two measurements, the two experimental series exhibit low dispersion around their respective linear fits, indicating a highly reproducible capacitive response for the analyzed sample.

Figure 5(b,c) presents the residuals associated with the linear fit in the depletion region for the measurements at 10 and 5 kHz, respectively. In both cases, the residuals remain centered around zero and show no significant deviations. The above confirms that the linearity observed in Figure 5(a) is preserved when assessing the quality of fit in the parameter-extraction intervals. Consequently, both the positive slope and the stability of the fit reinforce the assignment of n-type conductivity to our 2s TOS, in agreement with the previously obtained Hall-effect results.

Finally, Figure 5(d) quantitatively summarizes the information obtained from the analysis. For the fit over the entire interval, the slopes obtained were 4.030 × 10^12^ and 3.271 × 10^12^ F^–2^ V^–1^ for 10 and 5 kHz, respectively, with *R* ^2^ values of 0.99506 and 0.99918. When considering the selected depletion region, the calculated slopes were 4.175 × 10^12^ and 3.203 × 10^12^ F^–2^ V^–1^, with even higher fit coefficients, *R* ^2^ = 0.99953 and 0.99978. In all cases, the positive sign of the slope was unambiguously maintained. Based on these results, and using *ε*_*r*_(Cu_2_O) = 7.5 and an active area of 1.0 cm^2^, flat band potentials of −2.331 and −1.577 V vs Ag/AgCl were estimated for 10 and 5 kHz, respectively, as well as donor densities on the order of 10^18^cm^–3^, specifically 4.665 × 10^18^ and 5.747 × 10^18^ cm^–3^. The results of this electrochemical analysis independently corroborate the n-type nature previously identified by the Hall effect.

It is important to note that the apparent donor density values obtained through Mott–Schottky analysis, on the order of 10^18^ cm^–3^, are approximately 2 orders of magnitude higher than the free carrier concentration determined by the Hall effect, which is close to 2.3 × 10^16^ cm^–3^. This discrepancy stems from a physical difference between the two analytical methods. M-S analysis quantifies the total density of ionized donors in the space-charge region, including those whose electrons remain localized in deep traps or in states associated with grain boundaries and therefore do not participate in electrical transport. The Hall effect, on the other hand, only registers the mobile free carriers that actually contribute to conduction.

In a polycrystalline film like our TOS, grain boundaries act as trapping sites that immobilize a significant fraction of the electrons donated by oxygen vacancies and copper interstitials. Therefore, the density of carriers actually transported is lower than the density of ionized donors detected by capacitance. This difference is further accentuated by the slight frequency dispersion observed between the 5 and 10 kHz measurements, indicating deviations from ideal depletion behavior and consistent with the presence of interface states and traps in the film. However, the most valuable aspect is the agreement between both techniques in unequivocally identifying the n-type nature of our two-second TOS.

### 2.6 Nanostructural Characterization

Taken together, the metrological, optical, and electrical analyses consistently identify the 2 s sample as the most favorable copper-oxide TOS obtained under the present fast-annealing protocol. This film exhibits the smallest thickness uncertainty (t = 39 ± 6 nm), the highest average visible transmittance (*T*_avg_= 69% over 400–700 nm), and the largest optical bandgap among the series as estimated by the direct-transition Tauc analysis (Eg = 2.18 eV, compared with 2.14 and 2.01 eV for the 4 and 6 s films, respectively). Complementarily, the Zanatta approach yields *E*_*Gap*_^*Boltz*^ = 2.29 eV for the 2 s film and a relatively large energetic width (δE = 0.57 ± 0.03 eV), indicative of a polycrystalline material with a moderately defined absorption edge and a degree of structural disorder consistent with the observed optical transparency. Importantly, the marked inconsistency obtained when forcing an indirect-transition interpretation (*E*_*Gap*_^*Boltz*^≈ 0.019 eV) strongly suggests that optical absorption in the flagship film is governed by direct transitions, in line with a predominantly cuprite-like Cu_2_O phase.

Electrical transport further supports the selection of the 2s film as the flagship TOS. Hall measurements confirm n-type conduction across the series, and the 2s film provides the most favorable balance between transparency and conductivity, yielding the highest Haacke high-resolution figure of merit (Φ_H–HR_ = 0.1396, compared with 0.1161 and 0.0919 for the 4 and 6 s films). Consistently, it also exhibits the highest conductivity (σ = 2.92 × 10^–2^ S·cm^–1^) and an electron concentration of 2.3 × 10^16^cm^–3^. In light of this consolidated performance ranking, the nanostructural characterization below focuses on the 2 s film to address two practical questions: (i) whether its surface morphology is sufficiently compact and featureless at the SEM/TEM resolution to support integration in stacked device concepts, and (ii) whether its microstructure and diffraction fingerprints corroborate a polycrystalline Cu_2_O film with a discernible degree of structural order and a dominant single-phase cuprite signature.

Figure 6 summarizes the nanostructural assessment of the flagship 2 s Cu_2_O TOS using complementary electron microscopy. Panels (a–d) present SEM observations collected at increasing magnification to evaluate surface planarity and compactness at the micro- and nanoscale, which are key for assessing the film’s suitability for integration in functional device stacks. Panels (e–h) compile TEM-based evidence, including bright-field imaging, selected-area electron diffraction, and HRTEM, to establish the film’s polycrystalline character, its local degree of structural order, and a diffraction signature consistent with a dominant cuprite Cu_2_O phase.

**Figure 6:**
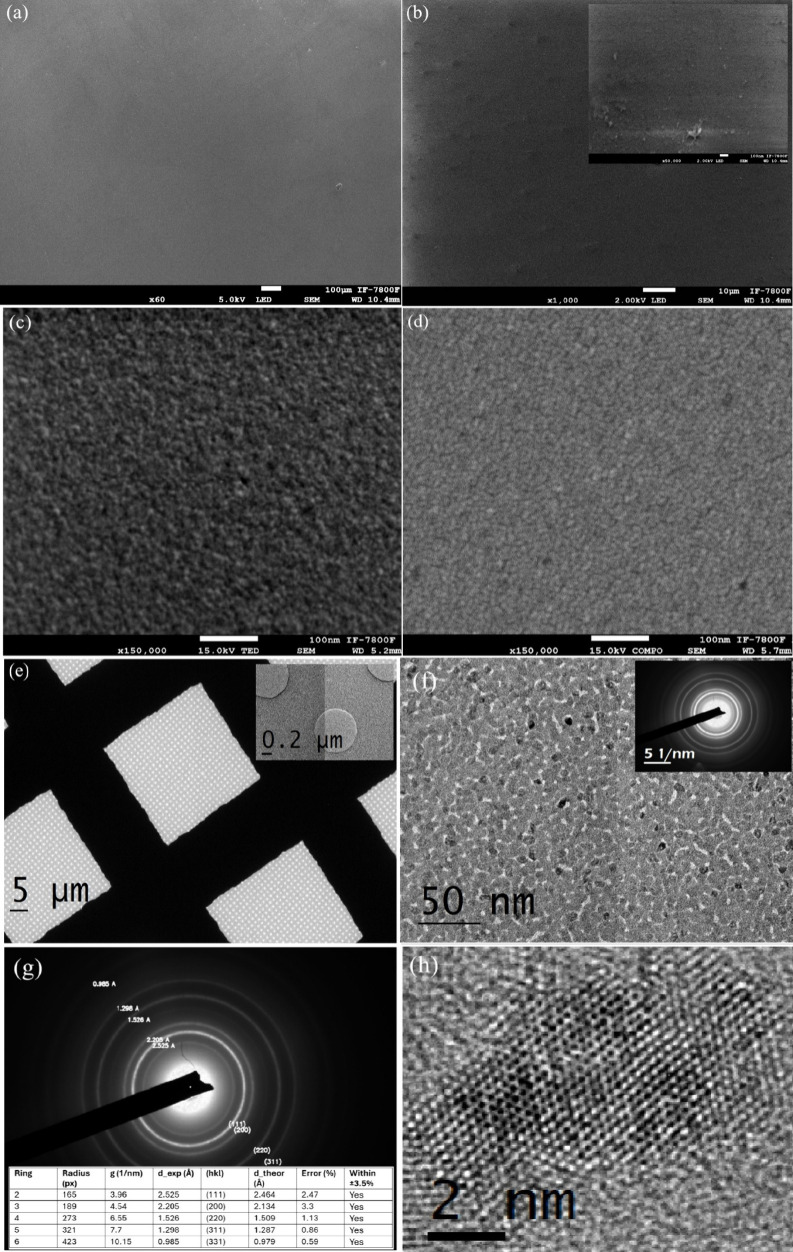
Nanostructural characterization of the flagship 2 s copper-oxide TOS obtained after low-power DC sputtering of Cu and brief annealing at 400 °C. (a–d) SEM images acquired at increasing magnification to evaluate surface continuity, planarity, and compactness across the film, including high-magnification views under topographic and compositional contrast modes. (e) Low-magnification TEM overview of the Quantifoil Ni support (∼1 μm hole size) used for specimen preparation, enabling large-area electron transparency. (f) Bright-field TEM image of the film, with the corresponding SAED pattern (inset) showing concentric rings characteristic of a polycrystalline oxide. (g) Indexed SAED pattern of the film together with the measured ring spacings, consistent with the cuprite Cu_2_O phase within the experimental resolution. (h) HRTEM image showing lattice fringes that corroborate the crystalline order at the nanoscale.

The meso- and microstructural morphology of the 2 s TOS was examined by progressively increasing magnification over the same region. Figure 6(a) shows a low-magnification secondary-electron (SE) micrograph acquired with the low-energy detector (LED) at 60×. At this mesoscopic scale, the film appears continuous and highly uniform, with no evident macroscopic defects such as cracks, delamination, or thickness nonuniformities, consistent with a compact oxide layer formed after the brief 400 °C thermal oxidation step.

Figure 6(b) presents an SE-LED image at 1,000× recorded in the same area, where an isolated particulate feature is observed. Given that this specimen had already undergone optical and electrical handling, this feature is most plausibly attributed to local debris introduced during manipulation rather than to a growth-related defect. Importantly, aside from this localized particle, the surrounding surface retains the same smooth and homogeneous appearance observed in Figure 6(a). At this magnification, faint charging-related contrast becomes noticeable, as expected for oxide films on insulating fused silica and consistent with the moderate conductivity reported for these samples. The inset in Figure 6(b) (50,000×, SE) further indicates that the surface remains compact and largely featureless in SE contrast, with no faceted grains resolvable at this scale. This observation suggests that either the characteristic lateral grain size is below the SE-resolvable range under the chosen conditions, or that the topographic contrast associated with grain relief is intrinsically weak for this dense film.

To probe the microstructure beyond SE topographic contrast, we subsequently acquired high-magnification images using complementary detection modes. Figure 6(c), recorded at 150,000× using the transmission electron detector (TED) in SEM-STEM bright-field configuration, reveals a fine granular contrast consistent with a nanocrystalline, densely packed microstructure rather than a truly featureless continuum. In parallel, the compositional/backscattered mode in Figure 6(d) (COMPO, 150,000×) provides clearer contrast variations across the field of view, which can arise from crystallographic channeling effects in polycrystalline materials (i.e., electron channeling contrast) and enables the emergence of grain-like domains while preserving an overall compact coverage. Notably, because the film is expected to be dominated by a single Cu–O phase, the COMPO contrast is not consistent with strong chemical/Z-contrast segregation (which would require markedly different average atomic numbers across the field), and is instead more plausibly attributed to orientation-dependent backscattering. Across this magnification sequence, no intermediate stage shows evidence of morphological degradation or loss of uniformity. Instead, the combined SEM contrast modes support that the low-power sputtering plus brief annealing route yields a continuous film with device-relevant planarity at the microscale, while already hinting at an underlying fine-grained polycrystalline texture that is later confirmed by TEM/SAED and HRTEM analyses.

Figure 6(e) shows the TEM support used for the nanostructural characterization, namely a Quantifoil holey film mounted on a 300-mesh Ni grid, consisting of an orthogonal array of circular apertures (nominal hole diameter 1.2 μm, pitch 1.3 μm). This support provides large electron-transparent windows that enable imaging and diffraction from regions where the film spans free-standing areas. The inset highlights one representative aperture; a faint contrast consistent with the presence of the transferred Cu-oxide layer can already be discerned across the opening, indicating that the specimen is sufficiently thin and continuous to allow TEM and SAED acquisition on suspended regions rather than on the support.

Figure 6(f) presents a TEM overview of the 2-s TOS on a suspended region, where the film appears continuous at the 50 nm scale while exhibiting a clear granular contrast. The contrast is spatially pervasive and lacks large featureless areas, which is consistent with a densely packed nanocrystalline microstructure instead of an amorphous continuum. From direct visual inspection of the grain-like contrast domains, the characteristic crystallite size is on the order of only a few nanometers, typically around 6 nm with a broad dispersion (roughly within the ∼ 2–10 nm range), as expected for a fine-grained polycrystalline layer. The inset SAED pattern associated with the same region displays well-defined concentric rings rather than a diffuse halo, confirming crystallinity and indicating that many randomly oriented nanocrystals contribute simultaneously to the diffracted intensity. In combination with the SEM contrast sequence in panels (a–d), which already hinted at a fine-grained texture emerging at high magnification while preserving overall planarity, the TEM overview reinforces that the low-power sputtering plus brief annealing route yields a compact, continuous film that is nanocrystalline in nature.

Figure 6(g) shows the SAED ring pattern acquired from a representative region of the 2-s TOS. The diffraction spacings were extracted by radial averaging of the 2D intensity distribution (masking the beam-stop sector) and subsequent peak detection on the resulting intensity-vs-radius profile. Ring radii *r*(in pixels) were converted to reciprocal-space magnitudes using the calibrated sampling of 0.024 (1/nm) per pixel, i.e., |*g*| = *r* × 0.024­(1/nm), and the corresponding interplanar spacings were obtained from *d* = 1/|*g*| (reported in Å). The quantified *d*-spacings for rings 2–6 are summarized in the inset table of Figure 6(g) and can be indexed to the cubic cuprite series as (111), (200), (220), (311), and (331), with deviations below ∼ 3.5% relative to the theoretical values. Using these *d*-spacings, the corresponding cubic lattice parameter estimated from individual rings clusters around ∼ 4.3 Å, which is consistent within experimental uncertainty with the reported lattice constant of cuprous oxide (Cu_2_O, space group *Pn3̅m*, ICDD PDF No. 05–0667; often cited as JCPDS 05–0667).^60^ Overall, the absence of extra rings beyond the indexed series supports a dominant single-phase cuprite-type diffraction signature at the level of SAED detectability.

Figure 6(h) presents a HRTEM image of an individual nanocrystallite within the film, where the periodic lattice-fringe contrast confirms local crystallinity. It is important to note that the bright and dark motifs in HRTEM are primarily phase-contrast features arising from the interference of transmitted and diffracted beams; thus, they could be interpreted as lattice fringes and projected atomic-column periodicities. Using the 2 nm scale bar as reference, the dominant fringe spacings are on the order of a few Å, consistent with the interplanar distances extracted from the SAED rings in Figure 6(g) (notably those associated with the low-index cuprite reflections). In this way, the reciprocal-space (ring) spacings and the real-space lattice periodicities provide mutually consistent evidence that the film is composed of nanocrystalline domains whose diffraction signature is dominated by cuprite-type Cu_2_O. Together with the TEM overview in Figure 6(f) showing pervasive granular contrast and the well-defined SAED rings in Figure 6(g), the HRTEM image in Figure 6(h) closes the structural loop by linking the film’s fine-grained morphology to a coherent crystalline periodicity compatible with a single cuprite-like phase.

### 2.7 X-ray Photoelectron Spectroscopy (XPS)

To identify the surface elements of our TOSs, we performed X-ray Photoelectron Spectroscopy (XPS) analysis. Figure 7 shows the survey spectrum recorded for the 2s sample. Two spectra are shown in this figure. The first corresponds to the film as it was introduced into the XPS vacuum chamber and represents the sample’s initial surface condition (black spectrum). The second spectrum was obtained after a surface-erosion process using ion sputtering, performed for 5 min with a 1 kV, 1 μA Ar^+^ beam over an area of 1 × 1 mm^2^ (red spectrum). The erosion process aims to distinguish the contributions from species in the outermost surface layer from those in the slightly subsurface region of the sample.

**Figure 7:**
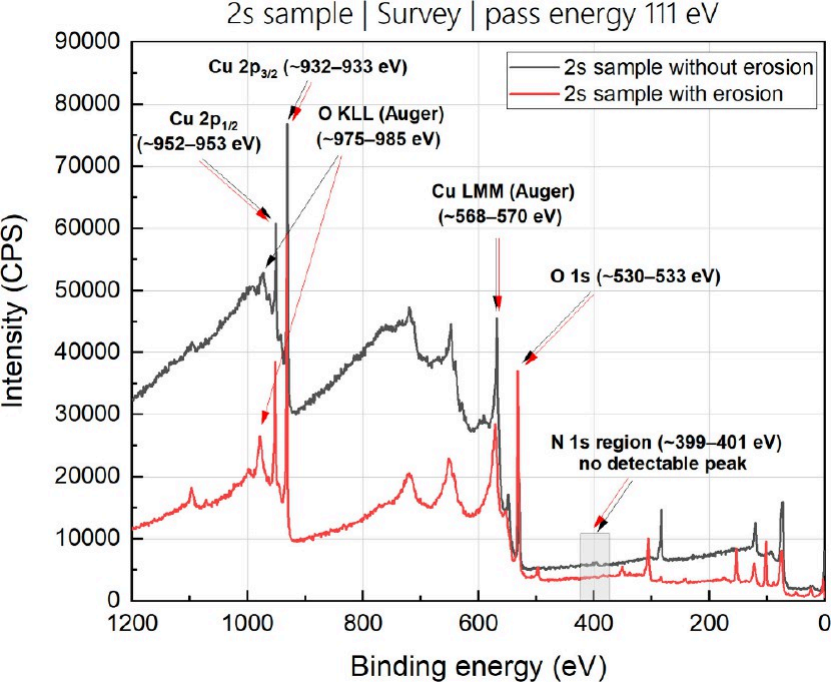
XPS spectrum recorded in survey mode for the 2s sample. Two spectra are shown, corresponding to the initial surface of the film as introduced into the analysis system (black spectrum) and to the same sample after a surface erosion process by sputtering with Ar^+^ ions for 5 min using 1 kV and 1 μA over an area of 1 × 1 mm^2^ (red spectrum). The characteristic signals associated with the Cu 2p doublet (∼932–933 and ∼ 952–953 eV), the Auger O KLL (∼975–985 eV) and Cu LMM (∼568–570 eV) transitions, as well as the O 1s level located around 530–533 eV, are identified in the spectrum. The arrows indicate the presence of these contributions in both spectra before and after the erosion process. The region corresponding to the N 1s level (∼399–401 eV), highlighted in a shaded box, shows no detectable signal within the analysis’s sensitivity limit.

The survey spectrum in Figure 7 clearly reveals that the dominant signals correspond to copper and oxygen. In the high-binding-energy region, the characteristic peaks of the Cu 2p doublet are identified. The most intense contribution corresponds to the Cu 2p_3/2_ transition located around 932–933 eV, while the Cu 2p_1/2_ component appears in the 952–953 eV region. Both transitions are indicated in the figure by black and red arrows, showing that the signals appear in both the spectra obtained before and after erosion.

In this same energy region, the signal corresponding to the Auger O KLL transition, located approximately between 975 and 985 eV, is also observed and is likewise marked by arrows in both spectra. Additionally, in the intermediate-energy region, a well-defined contribution associated with the Auger Cu LMM transition is observed around 568–570 eV. These signals are clearly indicated by black and red arrows, showing their presence in both the spectrum of the initial surface and that obtained after erosion.

The oxygen-associated signal is identified by the O 1s peak located approximately between 530 and 533 eV. This peak is clearly visible in both spectra and is indicated by arrows in the figure. Comparing the black spectrum corresponding to the initial surface with the red spectrum obtained after erosion reveals that the predominant peaks associated with copper and oxygen remain at equivalent energy positions. However, the relative intensity of the O 1s signal increases appreciably after sputtering, a behavior discussed in more detail in the high-resolution oxygen spectra of Figure 9.

Finally, in the region corresponding to the N 1s level, located approximately between 399 and 401 eV, no detectable peak is observed in either spectrum. This region is highlighted in Figure 7 with a gray shaded box to facilitate its identification. The absence of a signal in this energy range indicates that nitrogen is not detected within the XPS analysis’s sensitivity limit, either at the film’s initial surface or after the erosion process. To confirm this result, a high-resolution spectrum for this electronic level was also recorded, which showed no detectable signal.

To analyze in greater detail the electronic states associated with adventitious carbon and copper in the 2s sample, Figure 8 presents high-resolution spectra of the C 1s (left panel) and Cu 2p (right panel) levels. Both panels show the spectra recorded before (black) and after (red) surface erosion.

**Figure 8:**
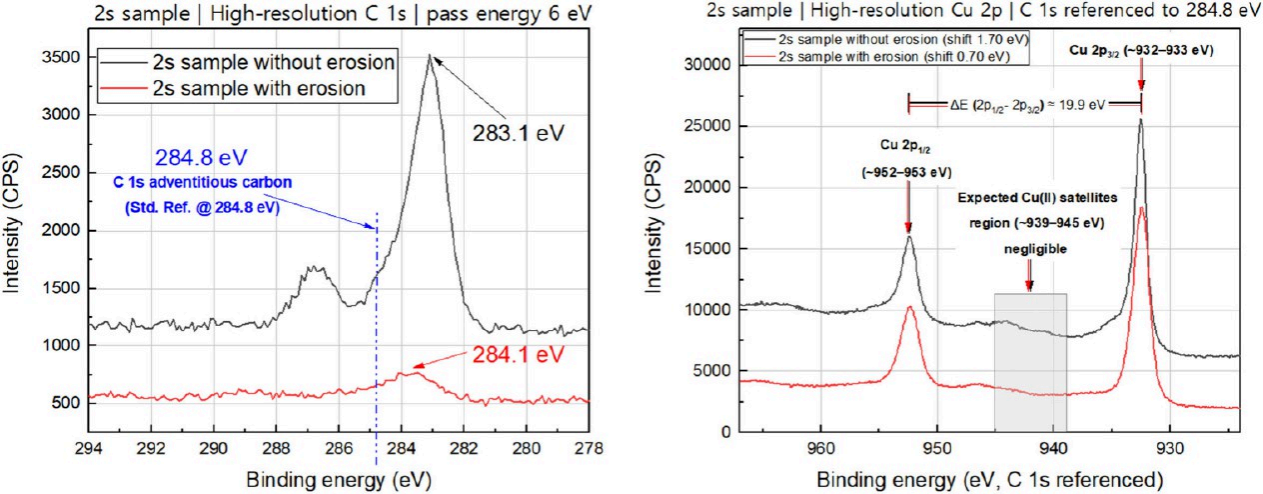
High-resolution XPS spectra recorded for the 2s sample. The left panel shows the C 1s level, used as an energy reference via adventitious carbon at 284.8 eV, indicated by a dashed vertical line. The spectra correspond to the film’s initial surface (black) and to the same sample after surface erosion (red). The right panel shows the Cu 2p doublet, in which the Cu 2p_3/2_ (∼932–933 eV) and Cu 2p_1/2_ (∼952–953 eV) components are identified, with an energy separation of 19.9 eV. The region where satellites associated with Cu­(II) species typically appear (∼939–945 eV) is indicated by a shaded box and shows no appreciable contributions.

The high-resolution C 1s spectrum was used as a reference for the energy correction of the XPS spectra. In this panel, the dashed blue vertical line marks the position of 284.8 eV, a value adopted for adventitious carbon and used as a conventional reference to compensate for surface charge shifts.^61^ The reason for explicitly indicating this energy is that the experimental maximum of the spectrum does not coincide directly with it. In the uneroded sample, the main maximum is located around 283.1 eV, while in the eroded sample, the observed contribution appears around 284.1 eV. Consequently, the black spectrum requires a shift of 1.7 eV, while the red spectrum requires a shift of 0.7 eV, as indicated later in the analysis of the Cu 2p doublet.^61^ The difference between the two shifts reflects that the electrostatic environment of the surface is not identical before and after erosion. This is attributed to the initial presence of surface contaminants and adsorbed species, which modify the charge state during measurement and are partially removed during the erosion process.^62^

The high-resolution spectrum corresponding to the Cu 2p doublet for the 2s sample before and after erosion is shown in the right panel of Figure 8. In both cases, the contributions associated with Cu 2p_3/2_, located around 932–933 eV, and Cu 2p_1/2_, located at 952–953 eV, are clearly distinguishable, with an energy separation of 19.9 eV between the two components. An important aspect of this result is that, after applying the charge correction derived from the analysis of the C 1s level, corresponding to 1.7 eV for the spectrum without erosion and 0.7 eV for the spectrum with erosion, the two doublet signals are aligned at the same energy positions in both spectra. The fact that, after applying this energy correction, the positions of Cu 2p_3/2_ and Cu 2p_1/2_ coincide exactly in both spectra constitutes an internal validation of the adventitious carbon-based energy referencing and confirms that the applied adjustment adequately describes the charge shift present in each surface condition.^61,62^ Furthermore, in the region approximately between 939 and 945 eV, indicated in the figure by a shaded gray box, no intense satellites attributable to Cu­(II) species are observed, but rather a negligible contribution.

An additional element that reinforces the assignment of the chemical state of copper comes from the Auger Cu LMM signal observed in the survey spectrum of Figure 7. The combination of the binding energy of the Cu 2p_3/2_ level with the kinetic energy of the Auger Cu LMM transition allows the evaluation of the well-known modified Auger parameter, whose estimated value from the energy positions observed in this work is around 1849 eV, consistent with a chemical environment dominated by Cu­(I) in Cu_2_O. This parameter has the advantage of being essentially independent of surface charge effects, since if the spectrum is shifted by charging, the binding energy increases, while the kinetic energy decreases by an equivalent amount, so their sum remains practically constant. In this regard, the consistency between the position of the Cu 2p doublet, the Cu LMM signal, and the absence of intense satellites attributable to Cu­(II) reinforces the conclusion that the analyzed film is dominated by a single phase based on Cu_2_O.

Although the analysis of the Cu 2p doublet and the modified Auger parameter clearly establishes that copper is predominantly in the Cu­(I) state, these results alone do not directly identify the point defects responsible for possible deviations from the ideal stoichiometry of Cu_2_O. In particular, the absence of signals attributable to Cu­(II) species and the stability of the Cu­(I) state suggest that the charge compensation mechanisms in the crystal lattice are not dominated by copper vacancies (V_Cu_). In this context, a detailed analysis of the oxygen contribution is essential to explore the possible presence of defects associated with the oxygen sublattice.

Figure 9 presents, in the left panel, the high-resolution spectrum corresponding to the O 1s level recorded before surface erosion (black spectrum) and after erosion (red spectrum). In this panel, a dashed blue line indicates the region associated with lattice oxygen in Cu_2_O, located approximately between 530 and 531 eV, and a dashed purple line indicates the region between 531.5 and 533 eV, where contributions associated with hydroxyl groups, adsorbates, and defect-related oxygen are located.

**Figure 9:**
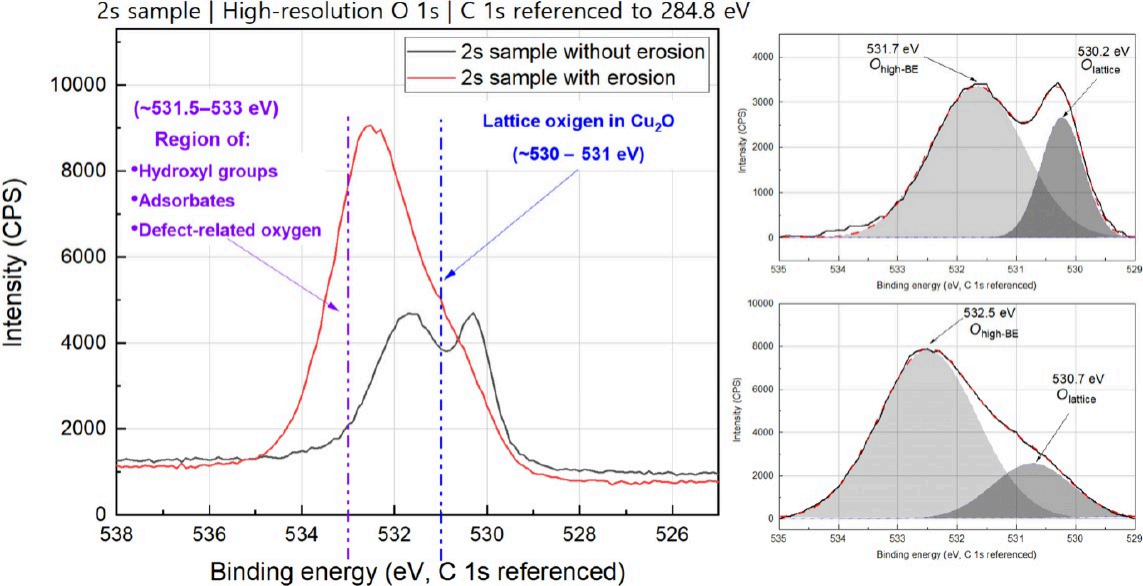
High-resolution XPS spectra corresponding to the O 1s level for the 2s sample. The left panel shows the spectra recorded before surface erosion (black) and after Ar^+^ sputtering (red), referenced to the C 1s level at 284.8 eV. This panel indicates two characteristic regions of the O 1s spectrum: the region associated with lattice oxygen in Cu_2_O (∼530–531 eV), indicated by a dashed blue line, and the region of higher binding energy (∼531.5–533 eV), marked with a dashed purple line, where contributions related to surface species or oxygen associated with defects typically appear. The upper and lower right panels show the deconvolutions of the O 1s spectrum for the sample without erosion and with erosion, respectively. In both cases, the experimental data were fitted using a Shirley background and Gaussian functions. The components obtained correspond to a lattice oxygen contribution (O_lattice) and a higher-energy binding contribution (O_high-BE). The black line represents the experimental spectrum, the dashed red line the overall fit, and the shaded areas the individual contributions obtained from the fitting process.

The upper and lower right panels of Figure 9 show the deconvolutions corresponding to the O 1s spectrum for the sample without erosion and with erosion, respectively. In both cases, the fit was performed using a Shirley background and Gaussian functions, yielding excellent agreement between the experimental data and the fitted curves. For the spectrum recorded before erosion, two main contributions are identified: one centered at 530.23 eV, attributed to lattice oxygen in Cu_2_O, and the other at 531.66 eV, associated with a higher-energy contribution frequently related to oxygen bound to defects, hydroxyl groups, or adsorbed species. The integrated areas obtained from the fit indicate that the higher-energy contribution accounts for approximately 73.4% of the total spectral area, while the contribution from lattice oxygen accounts for about 26.6%. The fit has an adjusted R-squared of 0.997 across 61 experimental points, indicating adequate reproduction of the experimental spectral shape.

After surface erosion, a clear redistribution of these spectral contributions is observed. In this case, the fit components are located at 530.73 eV for lattice oxygen and at 532.49 eV for the higher-binding-energy contribution. Quantitative analysis shows that the higher-energy contribution accounts for approximately 78.9% of the total area, while the component associated with lattice oxygen accounts for approximately 21.1%. The fit again exhibits excellent quality, with an adjusted R-squared of 0.999, confirming that the model used adequately describes the observed spectral profile.

The spectral distribution observed at the O 1s level suggests that the contribution in the higher-binding-energy region cannot be attributed solely to surface-adsorbed species. Although signals in the approximate 531–533 eV range are frequently associated with hydroxyl groups or adsorbed contaminants, several studies have indicated that this region can also include contributions related to oxygen associated with structural defects in metal oxides.^63^ In the present case, the fact that the higher-energy component remains clearly defined even after the surface erosion process indicates that this contribution does not originate from surface contaminants removable by sputtering. This observation, combined with the dominance of the Cu­(I) state evidenced in the analysis of the Cu 2p doublet and the absence of characteristic Cu­(II) satellites, suggests that the film is close to the characteristic stoichiometry of Cu_2_O, but with the presence of structural defects associated with the oxygen sublattice. In this context, the persistence of the higher-energy contribution in the O 1s spectrum is consistent with the presence of oxygen vacancies, which are widely reported in Cu_2_O deposited using physical techniques and can act as donor defects. Although XPS spectroscopy alone does not allow for unequivocal quantification of the concentration of these defects, the set of experimental evidence obtained in this work points to a scenario in which the Cu_2_O crystal lattice contains an appreciable fraction of deficient oxygen sites.^63^

## 3 Discussion

The consistent n-type conductivity observed in our Cu_2_O thin films is a remarkable result, considering that cuprous oxide is commonly reported as a p-type semiconductor. The conversion to n-type transport without intentional extrinsic doping suggests a fundamental modification of the defect chemistry, likely driven by the specific synergy between our low-power DC sputtering process and the subsequent thermal treatment in air.

First, the use of air as the sputtering gas introduces a unique chemical environment. Unlike conventional sputtering in pure Ar or Ar/O_2_ mixtures, deposition in air involves not only the presence of nitrogen but also a high partial pressure of oxygen during the initial metallic copper deposition stage. This chemical environment is expected to influence the early oxidation pathway of the deposited Cu and likely plays an important role in enabling its controlled conversion into Cu_2_O during the subsequent annealing step. However, despite the presence of nitrogen in air, the XPS analysis rules out its incorporation as the origin of the observed carrier polarity, since no detectable N 1s signal was found either before or after surface erosion, even after acquiring a high-resolution spectrum in that region. In parallel, the Cu 2p response, together with the absence of intense Cu­(II) shakeup satellites, indicates that the copper chemical state is dominated by Cu­(I), consistent with a Cu_2_O-based phase. Therefore, the observed n-type transport should not be attributed to nitrogen-related donor species, but rather to native donor-like defects generated and stabilized under this particular growth and oxidation route.

Second, the low sputtering power (20 W) is critical. At this regime, the kinetic energy of the sputtered Cu atoms and the ion bombardment on the substrate are minimized, significantly impacting the surface reaction kinetics. At such low sputtering energies, the incident Cu atoms have a reduced probability of overcoming the activation barriers required for immediate chemical reaction (chemisorption) upon impact. This lower mobility and energy decrease the likelihood of rapidly generating a cupric-like environment during the deposition stage and effectively bias the initial oxidation pathway toward *Cu* ^+^ instead of *Cu* ^2+^.

By favoring these lower oxidation states from the start, the system avoids the rapid formation of a CuO-like environment, which is typically associated with p-type behavior in higher-energy processes. This soft deposition promotes a specific initial microstructure with a high density of grain boundaries, observed by TEM, and a locally oxygen-deficient environment. During the short thermal treatment at 400 °C in air, oxidation occurs in a diffusion-limited regime. The XPS results support the view that oxygen-related donor defects are stabilized under these conditions. In particular, the O 1s deconvolution reveals a dominant high-binding-energy component centered at 531.66 eV before erosion and 532.49 eV after erosion, accounting for 73.4% and 78.9% of the total O 1s area, respectively, whereas the lattice-oxygen contribution represents only 26.6% and 21.1%.

Because the oxygen high-binding-energy contribution remains clearly defined after sputtering, it cannot be attributed solely to removable surface adsorbates and is instead consistent with defective oxygen environments, particularly oxygen vacancies (*V*_*O*_). Although XPS does not directly image interstitial species, within a Cu­(I)-dominated Cu_2_O matrix showing persistent signatures of oxygen-deficient local bonding and no evidence for nitrogen incorporation, copper interstitials (*Cu*_*i*_) become the most physically consistent complementary donor defect. In the defect chemistry of copper oxides, both *V*_*O*_ and *Cu*_*i*_ can act as donor-like centers and provide a coherent explanation for the electrons detected by Hall measurements, which show a consistent carrier concentration of 2.3 × 10^16^ cm^–3^.

The optical analysis further supports this defect-driven n-type behavior. The Zanatta model yields a δE = 0.57 ± 0.03 eV, indicating moderate structural disorder. This disorder is consistent with a polycrystalline lattice containing a significant concentration of point defects associated with oxygen deficiency and donor-like copper excess, namely *V*_*O*_ and *Cu*_*i*_, which do not degrade the transparency (∼70%) but are sufficient to shift the Fermi level toward the conduction band. The excellent agreement of our SAED patterns with the cuprite structure confirms that this n-type behavior is an intrinsic property of the Cu_2_O phase produced by this specific pathway, rather than a result of phase impurities or metallic clusters.

However, despite the structural consistency observed using TEM and SAED, the influence of extrinsic factors or subnanometer heterogeneities cannot be completely ruled out. Although our micrographs show no evidence of phase segregation or precipitates, the existence of copper metallic clusters with dimensions below the resolution limit of the techniques employed is theoretically possible. Such clusters, if present in an ultrafine volume fraction, could act as electron sources or modify the alignment of the energy bands, favoring n-type transport behavior without altering the diffraction patterns of the Cu_2_O matrix.

Furthermore, although the use of ambient air as the working gas and annealing atmosphere introduces inherent chemical complexity, the current XPS evidence does not support invoking nitrogen incorporation to explain the reproducible n-type polarity. Instead, the survey and high-resolution spectra are consistently described by Cu and O only, with no detectable N signal, a Cu­(I)-dominated Cu_2_O chemical state, and a persistent high-binding-energy O 1s contribution even after surface erosion. Accordingly, the remarkable consistency in our Hall effect measurements, in which all samples evaluated systematically exhibited n-type polarity, suggests that the reported methodology enables reproducible control of carrier type primarily through intrinsic donor-like defects associated with the oxygen sublattice, with *Cu*_*i*_ as the most plausible complementary donor species under this oxygen-deficient growth and oxidation pathway. This reproducibility is crucial, as it indicates that the phenomenon is a direct consequence of the growth and oxidation kinetics under the specific experimental conditions of our processing route.

The long-term stability of this defect configuration warrants explicit discussion, given that Cu_2_O is often cited as a material that is susceptible to further oxidation in air. However, this susceptibility is essentially based on a thermodynamic argument and disregards the process’s kinetics. The conversion of Cu_2_O to CuO requires the diffusion of Cu cations throughout the solid, a mechanism experimentally confirmed using marker techniques, and is governed by an activation energy of 80–114 kJ/mol, directly measured between 700 and 1000 K by Maack and Nilius.^64^ Applying the Arrhenius equation, the oxidation rate at room temperature is seven to 11 orders of magnitude lower than at 400 °C, which, in practical terms, equates to time scales of between 10^3^ and 10^6^ years for detectable oxidation to occur. This analysis indicates that, once the films reach their functional state after annealing at 400 °C, the defect configuration becomes kinetically frozen at room temperature. Under these conditions, the oxygen vacancy sites and the Cu interstitials associated with the n-type character remain in a dynamically stabilized, nonequilibrium state, the relaxation of which to thermodynamic equilibrium would occur on geological time scales under ambient conditions. This interpretation is consistent with the findings of Zan Lian et al.,^65^ who, using high-precision molecular dynamics, show that oxygen remains kinetically trapped in Cu_2_O even under reducing electrochemical conditions designed to extract it, underscoring that oxygen diffusion in this network is slow at room temperature, regardless of the direction of the process.

This kinetic argument has a direct implication for the reproducibility of the n-type character reported in this work. Since the concentration of donor defects is fixed by the annealing conditions, that is, by the temperature, duration, and atmosphere, and cannot appreciably relax once the film cools, the electronic state of the films becomes deterministically dependent on the manufacturing protocol and no longer dependent on subsequent storage history. From this perspective, the consistency observed in Hall effect measurements, where the n-type polarity and carrier concentration are reproduced within the margin of experimental uncertainty over time, is a direct consequence of this kinetic freezing. In other words, the controlled oxidation at 400 °C establishes the Cu_2_O phase, fixes the concentration of donor defects, and permanently locks this configuration under ambient conditions, conferring upon the films a functional stability that exceeds their thermodynamically metastable character.

Finally, we present Table 3, which benchmarks our best-performing 2 s TOS against, to the best of our knowledge, the full set of experimental reports available in the literature where copper oxides are explicitly demonstrated as n-type transparent semiconducting or conducting oxides, either intentionally doped or undoped. For consistency, the table compiles thickness, average transmittance, resistivity, sheet resistance, and the Haacke High-Resolution figure(s) of merit reported or derived from the original works. Values marked with *^b^* correspond to sheet resistances calculated from the authors’ reported thickness and resistivity using *R*_*s*_ = ρ/*t*, whereas average transmittance values marked with *^c^* were estimated from spectra or graphical data when not explicitly provided.

**Table 3:** Literature Benchmark of Experimentally Reported n-Type Copper-Oxide Transparent Semiconducting/Conducting Thin Films, Including the Present Work^a^

Deposition Technique	Material	Thickness [nm]	Average Transmittance (%)	Resistivity [Ω·cm]	Sheet Resistance [Ω/□]	Φ_*H*–*HR*_ [Ω^–1/10^]	Charge Carrier Density [*e*/*cm* ^3^]	Charge Type	ref
Spray pyrolysis	CuO:Mn	220	32	28.6	1.30 × 10^6^ ^b^	0.0783	1.00 × 10^17^	N	^32^
Spray pyrolysis	CuO:Mn	232	13	25.1	1.10 × 10^6^ ^b^	0.0323	2.00 × 10^17^	N	^32^
Spray pyrolysis	CuO: Mn	238	13	23.4	8.00 × 10^5^ ^b^	0.0334	3.00 × 10^17^	N	^32^
PLD	Cu_2_O	556	30^c^	2.12 × 10^–3^	38.1^b^	0.2085	3.47 × 10^21^	N	^37^
ECD	Cu_2_O:Cl	300	--	48	1.6 × 10^6^	--	--	N	^66^
ECD	Cu_2_O:Cl	300	--	7	2.3 × 10^5^	--	--	N	^66^
Spin coating	CuO:Co	--	42	361.4	--	--	1.247 × 10^14^	N	^34^
Spin coating	CuO:Zr	456	25^c^	17.06	3.7 × 10^5^ ^b^	0.0694	5.059 × 10^18^	N	^67^
Spin coating	CuO:Zr	453	40^c^	37.89	8.4 × 10^5^ ^b^	0.1022	2.896 × 10^17^	N	^67^
ECD	Cu_2_O:F	600	--	--	--	--	9.25 × 10^17^	N	^29^
ECD	Cu_2_O:K	--	5^c^	--	--	--	3.2 × 10^17^	N	^31^
ECD	Cu_2_O:Cl	--	--	--	--	--	1 × 10^20^ ^b^	N	^69^
ECD	Cu_2_O	--	--	--	--	--	3.5 × 10^19^ ^b^	N	^70^
Sputtering	CuO:In	350	20	442	1.26 × 10^7^ ^b^	0.0390	8.27 × 10^16^	N	^35^
Sputtering	CuO:In	350	20	116	3.31 × 10^6^ ^b^	0.0446	2.02 × 10^17^	N	^35^
Sputtering	CuO:Al	350	20	498	1.42 × 10^7^ ^b^	0.0385	1.71 × 10^17^	N	^35^
Sputtering	CuO:Al	350	20	250	7.14 × 10^6^ ^b^	0.0413	8.42 × 10^17^	N	^35^
Sputtering	CuO	--	22	--	--	--	1.457 × 10^19^	N	^36^
Sputtering	Cu_2_O	39	69	34.20	8.76 × 10^6^	0.1396	2.3 ± 0.29 × 10^16^	N	This work

a The table compiles deposition technique, oxide composition (doped or undoped), thickness, average transmittance, resistivity, sheet resistance, charge-carrier concentration, and Haacke High Resolution figures of merit.

b Rs values were calculated from the reported *ρ* and *t* using Rs = *ρ*/t.

c Tavg values were estimated from the transmittance spectra or graphical data when not explicitly provided by the authors. All entries correspond to studies that explicitly report n-type conduction.

As summarized in Table 3, the highest figure of merit among n-type copper-oxide TOSs is achieved by PLD-grown single-phase Cu_2_O, reaching Φ_*H*–*HR*_ = 0.2085, enabled by its surprising very low resistivity (∼2.1 × 10^–3^Ω•*cm*) and correspondingly low *R*_*s*_(tens of Ω/□) despite a moderate transmittance (estimated ∼ 30%). Notably, our sputtered-and-rapidly oxidized Cu_2_O film ranks next, delivering Φ_*H*–*HR*_ = 0.1396 while maintaining a high optical transparency (*T*_*avg*_ ≈ 69%) and a reproducible n-type carrier concentration (∼2.3 × 10^16^ *cm* ^–3^). The following best-performing case corresponds to intentionally Zr-doped CuO prepared by spin coating, highlighting that most alternative routes rely on extrinsic dopants to stabilize n-type transport. In this context, the present low-power sputtering plus short air-annealing pathway positions our undoped Cu_2_O among the top-performing experimentally demonstrated n-type copper-oxide TOSs reported so far.

Although our films exhibit the second-best figure of merit among n-type Cu_2_O TOSs reported in the literature, it is important to acknowledge that their high sheet resistance (8.76 × 10^6^ Ω/□) and relatively low carrier density (2.3 ± 0.29) × 10^16^ cm^–3^ represent significant limitations for their direct integration into p–n junction devices. Specifically, such a high series resistance would reduce carrier injection efficiency at the junction, while the low carrier density leads to a comparatively wide depletion region, increasing the likelihood of interfacial recombination. In this regard, controlled extrinsic doping with cations such as In^3+^ or Al^3+^, whose effectiveness in increasing carrier density in Cu_2_O from ∼ 10^14^ to ∼ 10^17^ cm^–3^ has been demonstrated by sputtering,^35^ constitutes the most direct strategy for improving conductivity. As complementary approaches, adjusting the partial pressure of O_2_ during deposition would allow modulation of the oxygen vacancy concentration without resorting to extrinsic dopants, and postdeposition heat treatments in controlled atmospheres could optimize the balance between donor defect density and carrier mobility. The systematic exploration of these alternatives represents a priority direction for future work aimed at developing functional devices based on these films.

## 4 Conclusions

In this work, we present a reproducible methodology to synthesize single-phase, n-type Cu_2_O thin films, overcoming the intrinsic p-type tendency of copper oxides. By combining low-power (20 W) DC sputtering in an ambient air atmosphere with a brief thermal treatment at 400 °C, we produced transparent oxide semiconductors (TOS) with an average transmittance of ∼ 70% and a wide optical bandgap of 2.2 eV. XPS analysis further supports the formation of a Cu_2_O-based phase by showing that the films are chemically dominated by Cu and O, with no detectable N incorporation, a Cu­(I)-dominated chemical state, and no intense shakeup satellites associated with Cu­(II).

Exhaustive structural characterization via SEM and TEM confirmed the formation of single-phase, polycrystalline films. The microstructural analysis revealed a nanometric crystalline architecture with an average crystallite size of 6 ± 4 nm. This fine-grained morphology, coupled with the “soft” deposition kinetics, is fundamental to the stability of the n-type behavior. In addition, AFM analysis revealed a smooth surface topography, with average RMS roughness values ranging between 1.30 and 3.19 nm. Such low roughness is particularly advantageous for device integration, since it can promote sufficiently abrupt interfacial transitions in homo- and heterojunction p-n architectures, as well as in thin-film photovoltaic and transistor platforms. Electrically, the films exhibited a resistivity of 34.2 Ω·cm and a consistent electron concentration of (2.3 ± 0.29) × 10^16^cm^–3^.These values, together with the calculated figures of merit (FOM) and a surface topography well suited for the construction of electronically well-defined junctions, demonstrate that our TOSs possess the optoelectronic and interfacial quality required for integration into experimental functional devices.

Our findings suggest that the synergy between the chemical complexity of the air plasma and the low-energy deposition favors a metastable defect chemistry dominated by oxygen-related donor defects, particularly oxygen vacancies (*V*_*O*_), with interstitial copper (*Cu*_*i*_) as the most plausible complementary donor species under this growth route. This interpretation is reinforced by the XPS results, which rule out nitrogen incorporation as the origin of the carrier polarity and show a persistent high-binding-energy O 1s contribution even after surface erosion, consistent with defective environments associated with the oxygen sublattice.

By providing a clear route to n-type Cu_2_O, this study complements our previous report on p-type films^9^, and completes the materials-level platform required for the fabrication of all-copper-oxide p–n junction architectures. However, it is important to underscore that the practical realization of such junctions will require a careful reconciliation of the distinct deposition and annealing windows. Since the p-type and n-type responses depend on different processing environments and thermal budgets, future research must focus on developing stacking strategies that preserve the specific defect chemistry of each layer during sequential processing. Nevertheless, we believe that the successful growth of stable n-type Cu_2_O thin films through this simple and scalable approach marks an important step toward all-copper-oxide transparent electronics and optoelectronic prototypes.

## 5 Experimental Section

### 5.1 Substrate Selection and Cleaning Protocol

We used fused silica and Corning glass as substrates. Fused silica substrates were utilized for ultraviolet–visible-near-infrared spectroscopy (UV–vis-NIR), while glass substrates were employed for Hall effect measurements. A 3 mm diameter nickel grid covered with nitrocellulose was placed atop some silica substrates for nanostructural characterization by TEM.

The cleaning process for the fused silica and Corning glass substrates consisted of ultrasonic baths, each for 5 min at room temperature, in the following order: (i) trichloroethylene to remove grease from the substrate; (ii) methanol (CH_4_O) to eliminate trichloroethylene residues; (iii) acetone (C_3_H_6_O) to eradicate organic particles; and (iv) methanol again to remove acetone residues. Finally, we dried the substrates using high-pressure jets of carbon dioxide (CO_2_).^71^

### 5.2 DC Sputtering Deposition Protocol

Copper precursor layers were deposited using a desktop, single-target DC magnetron sputtering system (Zhengzhou CY Scientific Instrument Co., Ltd.; model CY-MSZ180-DC-Q). The instrument comprises a 304 stainless-steel vacuum chamber (cavity size Ø180 mm × 200 mm) equipped with a removable top cover and an omnidirectional observation window, and it is pumped by a rotary-vane backing pump coupled to a turbomolecular pump (nominal pumping speeds of 1.1 L s^–1^ and 600 L s^–1^, respectively). The system base pressure is specified as 1.0 × 10^–4^ Pa and vacuum is monitored with a composite gauge covering 10^–5^ to 10^5^ Pa. A 2-in. unbalanced magnetron cathode accommodates a 50.8 mm diameter target (thickness ≤ 3 mm) and is powered by a DC supply (rated 300 W). Substrates were mounted on a Ø100 mm stage.

A 99.9995% purity Cu target was used. Prior to deposition, the chamber was evacuated to a cleaning pressure of 7 × 10^–6^ Torr (9.33 × 10^–4^ Pa). The target was then presputtered for 10 min with the shutter closed at 50 W to remove surface contaminants and stabilize the discharge.^72^ After presputtering, the DC power was reduced to 20 W and the working gas was set to atmospheric air (rather than Ar), composed primarily of N_2_ (78.084%), O_2_ (20.946%), Ar (0.934%), and CO_2_ (0.033%), with trace Ne (18.18 ppm), He (5.24 ppm), Kr (1.14 ppm), Xe (0.087 ppm), H_2_ (0.5 ppm), CH_4_ (2 ppm), and N_2_O (0.5 ppm),^73^ to reach a process pressure of 2 Pa (approximately 1.5 × 10^–2^Torr). Depositions were performed by opening the shutter for 2, 4, or 6 s. The target-to-substrate distance was fixed at 3 cm, and the target and substrate were aligned coaxially such that their geometric centers coincided. Square substrates with lateral dimensions of 1 cm were employed.

### 5.3 Thermal Oxidation Process

It is widely reported in the literature that when thermal energy is applied to thin films of ultrapure copper, the copper atoms on the film’s surface react with oxygen in the surrounding atmosphere to form different copper oxides.^60,74–79^ The temperature provides the energy necessary for the copper atoms to bond with the oxygen atoms. The activation energy for the oxidation of Cu to Cu_2_O is high in the low-temperature range.^80^ On this matter, several research groups have shown that temperatures between 150 and 250 °C favor the formation of single-phase Cu_2_O.^75,76^ However, Y. Zhu et al. discuss that the activation energy for the oxidation of Cu to Cu_2_O becomes very low, or even negative, only above 600 °C.^80^ Therefore, it is possible to assume that it is not strictly necessary to remain below 250 °C to achieve the formation of Cu_2_O from pure Cu thin films.

In this sense, it is essential to mention that the final characteristics of the Cu films after being subjected to the thermal oxidation process are closely related to the process parameters itself, such as the heating ramp, the cooling ramp, the atmosphere type, the maximum temperature of the thermal oxidation process, and, of course, the time the film remains at that temperature and atmosphere. In other words, each thermal oxidation parameter will influence the oxidized copper films’ microstructural, optical, and electronic properties.

From the above, this work explores the influence that rapid oxidation at 400 °C has on the final characteristics of our sputtered copper films. Figure 3(a) shows the characteristics of our proposed short-time annealing process. The samples were placed in an oven, and they remained inside during the rise and fall of temperature. These treatments were carried out in an air atmosphere using a PID-controlled resistance oven.

### 5.4 Spectrophotometric Transmittance Evaluation

Spectrophotometric transmittance measurements were performed at normal incidence using an Agilent Cary 5000 UV–vis-NIR spectrophotometer with a deuterium arc lamp dedicated to the UV section. All reported spectra have a resolution of 1 nm, and the spectral range studied spans from 175 to 1200 nm. The instrument performs a lamp change and a detector change at 350 and 800 nm, respectively.

### 5.5 Electrochemical Mott–Schottky Characterization

For Mott–Schottky capacitance analysis, a potentiostat/galvanostat Autolab PGSTAT 302N was used. The electrochemical cell was a classical three-electrode arrangement with Ag/AgCl as the reference electrode, a large-area graphite sheet as the counter electrode, and the sample ITO/Cu2O as the working electrode. The electrolyte was prepared from distilled water containing 0.1 M Na2SO4 (Merck reagents). The tests were performed at constant frequencies of 10 kHz and 5 kHz. Before the M-S tests, the sample was immersed for 20 min to allow stabilization.

### 5.6 Hall Effect Characterization

We used an Ecopia Hall Effect Measurement System (HMS-3000 Ver 3.51.5) with the Van der Pauw method to determine the conductivity, carrier type, and carrier concentration.^81^ The system is equipped with a permanent magnetic field of 0.54 T. This setup allows for precise measurements of the electrical properties of the thin films, including the determination of the Hall coefficient, carrier mobility, and resistivity. Measurements were conducted only at room temperature.

### 5.7 Nanostructural and Surface Characterization

The surface and nanostructural characterizations were conducted using Scanning Electron Microscopy (SEM) and High-Resolution Transmission Electron Microscopy (HRTEM). SEM analysis was conducted on all substrates, including the nickel grid, with a Schottky JEOL-JSM-7800F field-emission high-resolution SEM. The operating voltages ranged from 1 to 15 kV, and the resulting SEM images were processed with ImageJ software.^82,83^ For HRTEM characterization, the films on the nickel grids were analyzed using a JEOL JEM-2010F FasTEM microscope at 200 kV. The HRTEM images were taken with a CCD camera and processed using the GATAN digital micrograph system version 3.7.0.

### 5.8 XPS Characterization

X-ray photoelectron spectroscopy (XPS) analyses were performed on a PHI 5000 VersaProbe II Scanning XPS Microprobe system, operated under ultrahigh vacuum conditions (<7.5 × 10^–10^ Torr) and equipped with a monochromated Al Kα source (hν = 1486.6 eV), a 200 μm diameter X-ray beam, and a multichannel detector analyzer. Spectra were acquired at a 45° angle to the surface normal. To explore the subsurface region and attenuate the contribution of surface-adsorbed species, samples were subjected to ion etching with Ar^+^ for 5 min, using 1 kV and a current density of 0.1 μA mm^–2^. Analyses were performed on both the initial and postetching surfaces.

## Data Availability

The data that support the findings of this study are available within the article.
